# Analysis of matched primary and recurrent *BRCA1/2* mutation-associated tumors identifies recurrence-specific drivers

**DOI:** 10.1038/s41467-022-34523-y

**Published:** 2022-11-07

**Authors:** Jennifer B. Shah, Dana Pueschl, Bradley Wubbenhorst, Mengyao Fan, John Pluta, Kurt D’Andrea, Anna P. Hubert, Jake S. Shilan, Wenting Zhou, Adam A. Kraya, Alba Llop Guevara, Catherine Ruan, Violeta Serra, Judith Balmaña, Michael Feldman, Pat J. Morin, Anupma Nayak, Kara N. Maxwell, Susan M. Domchek, Katherine L. Nathanson

**Affiliations:** 1 Division of Translational Medicine and Human Genetics, Department of Medicine, Perelman School of Medicine at the University of Pennsylvania, Philadelphia, PA 19104 USA; 2 Experimental Therapeutics Group, Vall d’Hebron Institut d’Oncologia, Barcelona, Spain; 3 Hereditary Cancer Genetics Group, Vall d’Hebron Institut d’Oncologia, Barcelona, Spain; 4 Department of Medical Oncology, Hospital Vall d’Hebron, Universitat Autònoma de Barcelona, Barcelona, Spain; 5 Division of Surgical Pathology, Department of Pathology and Laboratory Medicine, Perelman School of Medicine at the University of Pennsylvania, Philadelphia, PA 19104 USA; 6 Abramson Cancer Center, Perelman School of Medicine at the University of Pennsylvania, Philadelphia, PA 19104 USA; 7 Division of Hematology-Oncology, Department of Medicine, Perelman School of Medicine at the University of Pennsylvania, Philadelphia, PA 19104 USA; 8 Basser Center for BRCA, Perelman School of Medicine at the University of Pennsylvania, Philadelphia, PA 19104 USA

**Keywords:** Cancer genomics, Cancer genetics, Breast cancer, RNA sequencing, Ovarian cancer

## Abstract

Recurrence is a major cause of death among *BRCA1/2* mutation carriers with breast (BrCa) and ovarian cancers (OvCa). Herein we perform multi-omic sequencing on 67 paired primary and recurrent BrCa and OvCa from 27 *BRCA1/2* mutation carriers to identify potential recurrence-specific drivers. *PARP1* amplifications are identified in recurrences (False Discovery Rate q = 0.05), and *PARP1* is significantly overexpressed across primary BrCa and recurrent BrCa and OvCa, independent of amplification status. RNA sequencing analysis finds two *BRCA2* isoforms, *BRCA2-201/Long* and *BRCA2-001/Short*, respectively predicted to be sensitive and insensitive to nonsense-mediated decay. *BRCA2-001/Short* is expressed more frequently in recurrences and associated with reduced overall survival in breast cancer (87 vs. 121 months; Hazard Ratio = 2.5 [1.18–5.5]). Loss of heterozygosity (LOH) status is discordant in 25% of patient’s primary and recurrent tumors, with switching between both LOH and lack of LOH found. Our study reveals multiple potential drivers of recurrent disease in *BRCA1/2* mutation-associated cancer, improving our understanding of tumor evolution and suggesting potential biomarkers.

## Abstract (web summary)

Carriers of pathogenic *BRCA1/2* variants have a higher risk of breast and ovarian cancers, which recur frequently. Here, the authors sequence primary and recurrent tumours of *BRCA1/2* mutation carriers, finding *PARP1* amplifications, differential *BRCA2* isoform usage, and discordant loss of heterozygosity that are associated with recurrence.

## Introduction

Pathogenic variants in *BRCA1/2* are the most common cause of hereditary breast and ovarian cancer, underlying 5–10% of breast and 20% of ovarian cancers^1,2^. BRCA1 and BRCA2 function independently to maintain genomic integrity, with both playing critical roles in the homologous recombination (HR) pathway of DNA repair^3^. The loss of BRCA1 or BRCA2 allows cells to accrue mutations and genomic rearrangements, which facilitate a transition to cancer^4^. Carriers of pathogenic germline variants in *BRCA1/2* have an increased risk of breast and ovarian carcinomas, along with prostate and pancreatic cancers^3,5^.

Women with pathogenic variants in *BRCA1*/*2* are diagnosed with high-grade serous ovarian carcinomas^6^. *BRCA1* mutation carriers are predisposed to hormone receptor-negative breast cancers, whereas *BRCA2* mutation carriers more commonly develop hormone-positive breast cancers^3^. Ovarian and triple-negative breast cancers tend to recur within a few years of diagnosis^6,7^. Recurrences are usually associated with therapeutic resistance, and both recurrent ovarian cancer and metastatic breast cancer are considered incurable. The standard of care in *BRCA1/2* mutation-associated ovarian cancers is platinum-based chemotherapy (cisplatin, carboplatin) as first line treatment, with poly (ADP-ribose) polymerase inhibitors (PARPi) used increasingly in both the maintenance and metastatic settings. For *BRCA1/2* mutation-associated breast cancer, systemic therapy is given as appropriate to the stage and receptor status of a patient’s disease. PARPi are employed for late-stage breast cancer, and the OlympiA trial has suggested benefit in the adjuvant setting^8–10^. Although tumors in *BRCA1/2* mutation carriers may initially respond to platinums and PARPi, many develop resistance over time. Preclinical studies have demonstrated that platinum and PARPi resistance are mediated by several of the same mechanisms, such as expression of MDR1 (ABCB1) efflux pumps or loss of DNA damage response regulators (TP53BP1, REV7, CHD4, and PARP1)^11–19^. However, in tumors from *BRCA1/2* germline mutation carriers, the most common identified resistance mechanism to platinums and PARPi are reversion mutations affecting the mutant *BRCA1/2* allele^11,17,20–26^.

Prior studies of matched primary and recurrent *BRCA1/2* mutation-associated breast and ovarian cancers have been limited by small numbers (3–8 patients) or focused only on tumors resistant to PARPi, not the entire breadth of frontline therapy and resistance^17,27–31^. At present, most *BRCA1/2* mutation carriers with breast cancer do not receive PARPi prior to their first recurrence. Therefore, acquired resistance mechanisms to conventional frontline breast and ovarian cancer therapies are largely unknown. In addition, clinical trials have evaluated the efficacy of immune checkpoint blockade on *BRCA1/2* mutation-associated breast and ovarian cancers, with variable objective response rates^32–39^. Characterizing expression of checkpoint proteins in primary and recurrent *BRCA1/2* mutation-associated tumors may help to identify markers of response to immunomodulatory therapies.

To address outstanding questions about cancer recurrence in *BRCA1/2* mutation carriers, we performed an analysis of paired primary and recurrent *BRCA1/2* mutation-associated tumors. The sample set consisted of 67 primary and recurrent tumors from 13 *BRCA1/2* germline mutation carriers with breast cancer and 14 *BRCA1/2* germline mutation carriers with ovarian cancer. We performed a combination of whole exome, targeted, and RNA sequencing on this cohort of specimens, in addition to multiplex immunofluorescent microscopy^40,41^. Our goal was to identify genetic, transcriptomic, and immune events that underlie acquired therapeutic resistance. We hypothesized that differences between paired tumors would indicate mechanisms of tumor evolution associated with post-treatment tumor recurrence in *BRCA1/2* mutation-associated cancers.

## Results

### Cohort selection and clinical characteristics

We studied matched primary and recurrent breast cancers from 13 subjects, nine *BRCA1* and four *BRCA2* pathogenic variant carriers (Table 1). All but one *BRCA2* mutation carrier with breast cancer were female. Nine of 13 primary breast tumors were estrogen receptor expression negative (ER-), predominantly triple-negative breast cancer (TNBC) with concordant ER- recurrences. We evaluated matched primary and recurrent ovarian cancer from 14 women, 10 *BRCA1* and four *BRCA2* pathogenic variant carriers. Most (9/14) primary ovarian tumors were diagnosed at stage III or IV. As treatment for their primary tumor, all patients received chemotherapy (Table 1). All 14 (100%) ovarian cancer patients received platinums and 4/14 (29%) received PARPi prior to a collected recurrence. Three of 13 breast cancer patients (23%) received platinums and a single breast cancer patient (1/13; 8%) received PARPi. Breast cancer patients received hormonal therapy and radiation (38 patients, 54%) more frequently than ovarian cancer patients (21 patients, 21%). Additional clinical and sequencing details for each tumor are documented in Supplementary Data 1.

**Table 1:** Characteristics of primary/recurrent patient cohort and tumors

		Breast	Ovarian	Total
Patients		13	14	27
Tumors	Primary	13	14	27
	Recurrent	18	22	40
	Total	31	36	67
Number of recurrences collected per patient	1	9	11	20
	>1	4	3	7
Germline mutation	*BRCA1*	9	10	19
	*BRCA2*	4	4	8
Estrogen receptor (ER) status of primary tumor	ER+	4	N/A	4
	ER-	9	N/A	9
Stage at diagnosis	I	2	1	3
	II	8	3	11
	III	2	8	10
	IV	0	1	1
	Unreported	1	1	2
Treatment received^a,b^	Platinums	3	14	17
	PARPi^c^	1	4	5
	Chemotherapy	13	14	27
	Hormonal therapy	5	3	8
	Radiation	7	3	10

^a^Treatments are limited to reflect the number of patients from whom a post-treatment recurrence was collected.

^b^Treatment groups are not mutually exclusive; most patients received multiple treatments.

^c^*PARPi* poly (ADP-ribose) polymerase inhibitor.

### Analysis of somatic mutations from whole-exome and targeted sequencing

We used whole-exome sequencing (WES) to assess the landscape of somatic mutations in 67 paired primary/recurrent tumors and matched germline samples from 27 *BRCA1/2* mutation carriers with breast or ovarian cancer. We also re-sequenced 44 tumors and 18 respective germline samples at high depth (>300 ×) using a custom targeted capture of 209 genes (Supplementary Table 1).

**Fig. 1:**
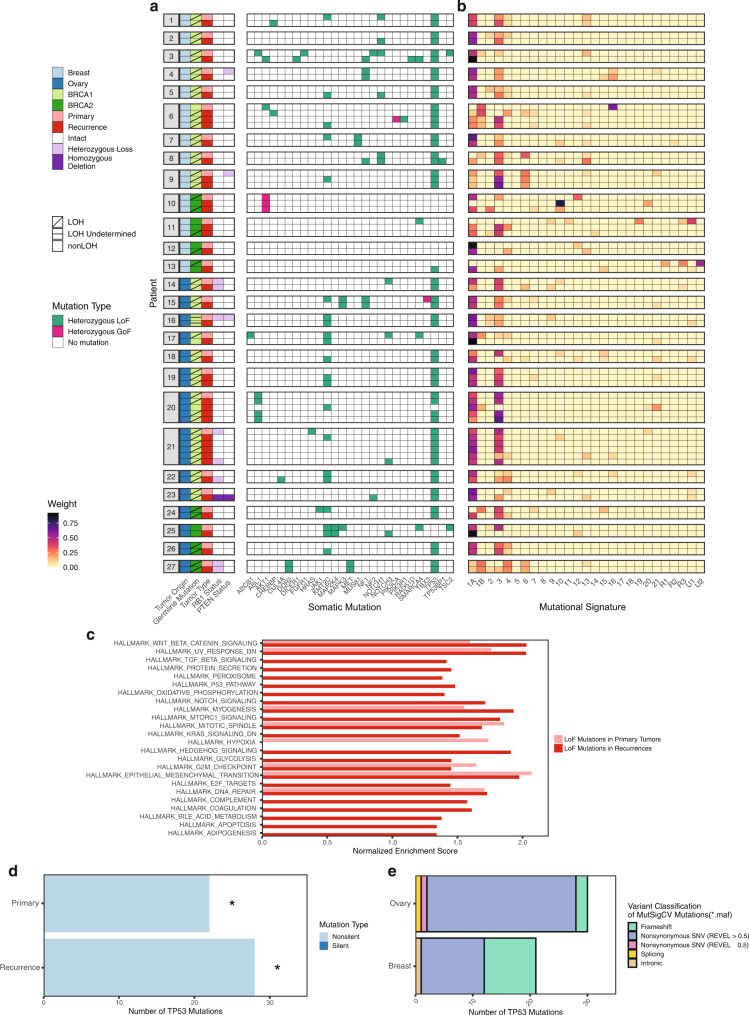
Integrated analysis of somatic mutations by whole-exome and targeted sequencing. **a** Phenotype, *BRCA1*/*2* allele-specific loss of heterozygosity (LOH), *RB1* and *PTEN* status, and somatic mutations found in 67 paired primary and recurrent tumors (from 27 patients) sequenced by whole-exome sequencing (WES, *n* = 67) and high-depth targeted sequencing (*n* = 44). Display is limited to genes with ≥1 mutation with alternative allele fraction (AAF) ≥ 0.05 from targeted sequencing and variants with AAF ≥ 0.05 from WES. Tumors are displayed in chronological order by patient, with the primary tumor at the top and latest recurrence at the bottom. LoF loss of function, GoF gain of function. **b** Mutational signatures derived from WES mutations. **c** Hallmark Gene Sets enriched in LoF mutations for primary and recurrent tumor groups (all FDR *q* < 0.25). **d** MutSigCV results for both primary and recurrent tumors (computed independently, *FDR *q* < 0.05 per MutSigCV results). **e** Variants contributing to MutSigCV results in **d**, by tumor type. SNV single-nucleotide variant.

We assessed individual gene variants with alternative allele frequency over 5% in oncogenes or tumor suppressors to identify whether they were shared across primary and recurrent tumors (Supplementary Data 2). The majority of the cohort (53/67 tumors; 80%) had loss of function (LoF) mutations in *TP53*. They were concordant between the primary tumor and recurrence(s) in all but one of the patients, when limited to alternative allele fraction (AAF) of ≥5%; several tumors had additional *TP53* mutations at AAF < 5% (Fig. 1a; Supplementary Fig. 1a–c and Supplementary Data 3). Patient 21 had two different pathogenic variants in *TP53*: p.C141R in the primary ovarian cancer and in her third and fifth recurrences; and p.P278S in her first, second, and fourth recurrences (Supplementary Data 3). Patient 13 had the same *TP53* mutation (p.E285K) at 1.2% AAF in the primary tumor and 32.9% AAF in the recurrence. Breast tumors without *TP53* mutations were mostly *BRCA2* mutation-associated and estrogen receptor positive (ER+) in origin. Five ovarian tumors from three patients (17, 20, 25) also lacked *TP53* mutations.

*NOTCH1* mutations were identified exclusively in breast tumors, including one primary tumor, two recurrences, and one matched pair of primary/recurrent tumors. *NF1* was mutated in both the primary and recurrence for one breast and one ovarian cancer, and in two breast cancer recurrences. One *BRCA2* mutation carrier (Patient 10) had an ER + primary breast tumor and two recurrences lacking *TP53* mutations; instead, the tumor appeared to be driven by the oncogenic *AKT1* E17K mutation^42,43^. Gain of function (GoF) mutations were identified in *PIK3CA* and *TBX3* in one breast and one ovarian tumor, respectively. We also noted pathogenic variants in *TP53BP1* (post-PARPi) and *PARP1*, each in a solitary *BRCA1* mutation-associated recurrence.

We next examined the global landscape of mutations by tumor. Mutational signatures were dominated by Signature 3 (*BRCA1/2* inactivating mutations and defective HR) and Signatures 1A/B (aging)^44^ (Fig. 1b). Sixteen of 27 (60%) tumors had mutations associated with Signature 4 (smoking)^44^. In paired tumor comparisons, tumor mutational burden (TMB) did not change from primary tumor to recurrence (Supplementary Fig. 1d and Supplementary Data 4). To investigate functional impact, we determined whether LoF variants accumulated in common pathways across the cohort. We ranked genes by prevalence of LoF variants within primary and recurrent tumor groups, then performed Gene Set Enrichment Analysis (GSEA) Preranked with Hallmark Gene Sets (Fig. 1c and Supplementary Data 5)^45^. LoF variants from recurrences were enriched for sixteen gene sets not found in primary tumor LoF variants (all FDR *q* < 0.25). These gene sets highlighted potential differences in metabolic processes (oxidative phosphorylation, glycolysis), apoptosis, TP53 signaling, and pathways regulating cell growth and identity (TGF*β*, Notch, MTORC1, KRAS).

To identify additional driver mutations in *BRCA1/2* mutation-associated tumors, we performed MutSigCV analysis on primary and recurrent tumor groups independently (Fig. 1d, e and Supplementary Data 6)^46^. *TP53* was the only significantly mutated gene within both tumor groups (FDR *q* < 0.05).

### Analysis of *BRCA1/2* loss of heterozygosity

**Fig. 2:**
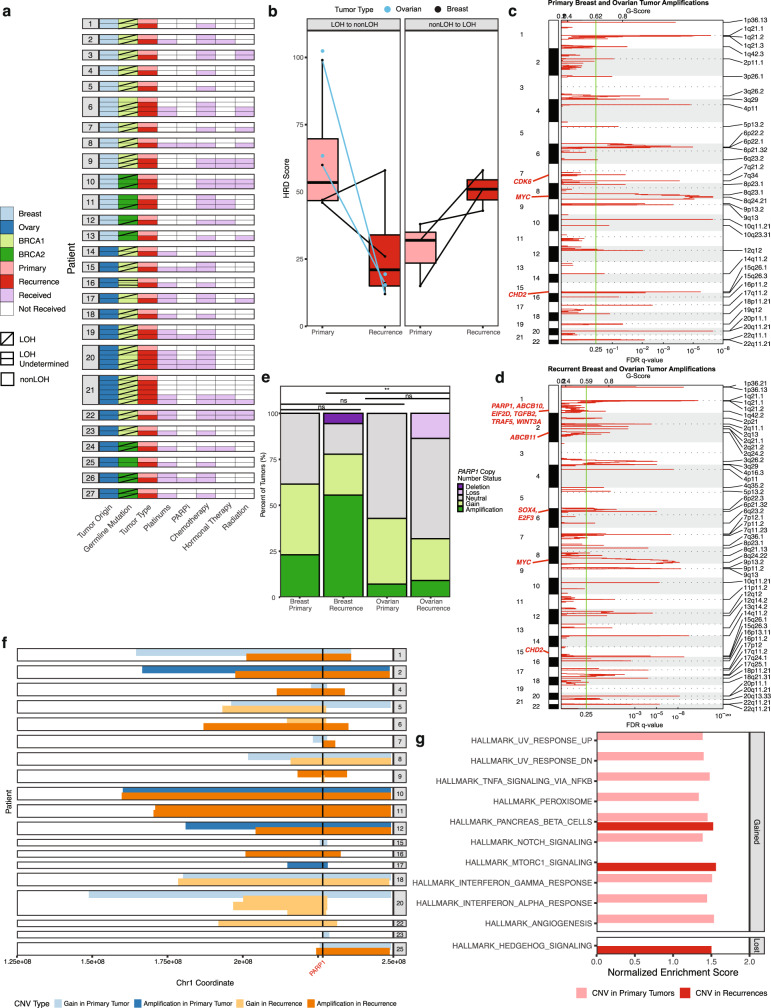
Genome-wide and gene-specific copy number variation. **a** Allele-specific loss of heterozygosity (LOH) of *BRCA1/2* in 67 paired primary and recurrent tumors, with treatment exposure. Tumors are displayed in chronological order by patient, with the primary tumor at the top and latest recurrence at the bottom. PARPi PARP inhibitor. **b** Comparison of Homologous Recombination Deficiency (HRD) score for tumors with a change in LOH status from primary to recurrence (*n* = 14 biologically independent samples, comprising 2 samples/patient from 7 patients). For one patient (Patient 6), who had multiple recurrences with LOH, the first recurrence is displayed. Boxplot elements are as follows: median, center line; box limits, first and third quartiles (spanning the IQR, interquartile range); whiskers, 1.5× IQR in each direction; outliers plotted individually. **c** GISTIC qplot for 90% confidence interval amplifications in all primary tumors (breast and ovarian). **d** GISTIC qplot for 90% confidence interval amplifications in all recurrences (breast and ovarian). For **c** and **d**, all highlighted genes have residual *q* ≤ 0.05. **e** *PARP1* copy number by tumor in primary/recurrent cohort. Groupwise differences in average copy number were determined by Kruskal–Wallis test, followed by Dunn’s test with Bonferroni correction (*α* = 0.05, **p < 0.01). NS not significant. **f** Segments of copy number gains and amplifications encompassing *PARP1*, by patient. For **e** and **f**, total copy number (Sequenza) was binned as follows: Deletion, CN = 0; Loss, CN = 1; Neutral, CN = 2–3; Gain, CN = 4–5; Amplification, CN ≥ 6. CNV copy number variation. **g** Hallmark Gene Sets enriched in primary and recurrent tumor gains and amplifications (CN ≥ 4, top) vs. losses and deletions (CN ≤ 1, bottom) (all FDR *q* < 0.25 per gene set enrichment analysis).

Using whole-exome and targeted sequencing, we assessed *BRCA1/2* allele-specific loss of heterozygosity (LOH). First, we assessed whether somatic mutations in *BRCA1/2* caused biallelic loss of function in our sample set of tumors (Supplementary Data 7 and Supplementary Data 8). We found pathogenic variants in the respective *BRCA1* or *BRCA2* genes in 20 of 67 tumors, all with very low alternative allele fraction (< 2.7%), suggesting that if real, these mutations only affected a minority of cells (Supplementary Data 8). Next, we used allele-specific copy number to determine whether tumors underwent LOH via copy number loss of their wild-type *BRCA1/2* allele^47^. Overall, 22/27 (81%) primary tumors and 33/40 (82%) recurrences demonstrated LOH (Fig. 2a). Most primary/recurrent tumor pairs had concordant *BRCA1/2* allele-specific LOH status (20/27 patients; 74%). Seven patients showed discordant LOH status when comparing their primary tumor to recurrence(s). Discordant tumor LOH status was more common in *BRCA2* mutation carriers, likely due to the lower prevalence of LOH within ER+ breast cancers^48^.

We observed discordant LOH status more frequently in breast cancers (five—38%) than in ovarian cancer patients (two—14%). Notably, three of four ER+ breast cancer patients in the cohort had discordant LOH in their tumors. We noticed overlap between patients with LOH transitions and those with *TP53* wild-type tumors (6/7 patients—86%). In three patients (6, 11, 12), the primary tumor did not have LOH, but first exhibited LOH upon recurrence (nonLOH to LOH, Supplementary Fig. 2a, b). Conversely, patients 10, 13, 17, and 20 showed the opposite effect: primary tumors exhibited LOH but had at least one nonLOH recurrence (LOH to nonLOH, Supplementary Fig. 3a, b). This “LOH reversal” was observed in two *BRCA2* mutation carriers with breast cancer, treated with non-platinum chemotherapy and radiation, and two *BRCA1* mutation carriers with ovarian cancer, both treated with platinums. In patient 20, mutational Signature 3 was also lost, reflecting the change in HR proficiency. For patient 10, Signature 3 was present in the primary tumor (with LOH) but not in the first recurrence (nonLOH) nor the final recurrence (again with LOH) (Fig. 1b). For patient 13, Signature 3 was observed in the recurrence without LOH, but not the primary with LOH. In patients 10, 17, and 20, nonLOH recurrences also had a lower HRD score compared to primary tumors with LOH, including decreases in all three individual HRD metrics used to compute the score (Fig. 2b and Supplementary Data 4). For all three tumors that first developed LOH upon recurrence, nonLOH primary tumors had a lower HRD score than recurrences (Fig. 2b). We did not find any associations between LOH discordance and treatment history.

### Assessment of genomic scarring and copy number variation

We next evaluated whether recurrent tumors showed more chromosomal instability and genomic scarring than primary tumors. Neither aneuploidy nor HRD scores (individual metrics or combined) were significantly different between tumor pairs overall, although some primary and recurrent tumors did have different scores (Supplementary Fig. 4a–e and Supplementary Data 4). To identify commonly amplified and deleted genes in primary and recurrent tumors, we performed GISTIC analysis, stratified by tumor type (Fig. 2c, d; Supplementary Figs. 4f, g and 5a–d; Supplementary Data 9)^49^. In a comparison of GISTIC segments between breast and ovarian tumors, we identified intersections (>50% overlap) for sixteen deletions and eight amplifications (Supplementary Data 9). As the tumor types exhibited similar copy number profiles, we combined all primary and all recurrent tumors to identify shared and distinct amplifications and deletions (Supplementary Data 10). Significant (90% CI and FDR *q* < 0.05) deletions were more similar between primary and recurrent tumors than significant amplifications (Jaccard similarity index of 0.20 vs. 0.06, Supplementary Data 10). Primary tumors had amplifications in *MYC* and *CHD2* (Fig. 2c). Recurrent tumors shared these amplifications and were also uniquely enriched for amplifications of *ABCB10*, another ABC transporter associated with chemotherapy resistance (Fig. 2d)^50^. Further, we noted recurrence-specific amplifications in *TGFB2, TRAF5, WNT3A, SOX4*, and *E2F3*, suggesting that recurrences may have increased dosage of growth and lineage signal pathway genes. In recurrences, unique deletions included *CASP9, CDKN2C*, *JUN, MUTYH*, and *RAD54L*, suggesting dysregulation of cell cycle, DNA damage response, and apoptosis (Supplementary Fig. 4g). In addition, our subtractive analysis also identified recurrence-private deletions in *KMT2C* and *MAD1L1*.

We found *PARP1* to be significantly (90% confidence interval, FDR *q* = 0.05) amplified in recurrences but not primary tumors (Fig. 2d). Given the use of PARPi for treatment in *BRCA1/2* mutation-associated tumors, we investigated further. Over half of tumors (35/67, 52%) in the cohort had somatic gains (copy number ≥4) or amplifications (copy number ≥6) in *PARP1* (Supplementary Data 11)*. PARP1* gains and amplifications were present in primary ovarian tumors (43%), recurrent ovarian tumors (32%), primary breast tumors (62%), and recurrent breast tumors (78%) (Fig. 2e). Seventy percent of all patients (19/27) in the cohort had a *PARP1* gain or amplification in at least one tumor (85% of breast patients, 57% of ovarian patients). Most of these patients (11/19, 60%) had a *PARP1* gain or amplification in both primary and recurrent tumors. Gains and amplifications tended to be focal and centered around the *PARP1* locus, rather than arm-level, underscoring a potential selection effect (Fig. 2f)^49^. We also evaluated *PARP1* copy number status in TCGA PanCancer cohorts of primary breast and ovarian tumors. We grouped TCGA tumors into *BRCA1/2* germline mutation-associated (*gBRCA1/*2) and HR wild-type (HR-WT) groups, as previously described (Supplementary Fig. 6a and Supplementary Data 12)^48,51^. Using the same methods for copy number analysis as utilized for the primary/recurrence cohort to analyze the TCGA Level 1 data, we found a high prevalence of *PARP1* gains and amplifications in both tumor types, with no differences based on *BRCA1/2* status (Supplementary Fig. 6b and Supplementary Data 11).

To assess copy number variation by pathway, we next performed GSEA Preranked on lists of genes ranked by prevalence of copy number gain or loss within each tumor group (Fig. 2g and Supplementary Data 5). Primary tumors were enriched for gains in gene sets for UV response, TNF*α* signaling via NF-*κ*B, IFN*α* and IFN*γ* response, and angiogenesis. Recurrent tumors were uniquely enriched for gains in MTORC1 signaling genes and deletions in Hedgehog signaling genes (consistent with our finding that recurrent tumors accumulate LoF mutations in Hedgehog signaling genes).

### Global transcriptomic analysis of primary and recurrent tumors

**Fig. 3:**
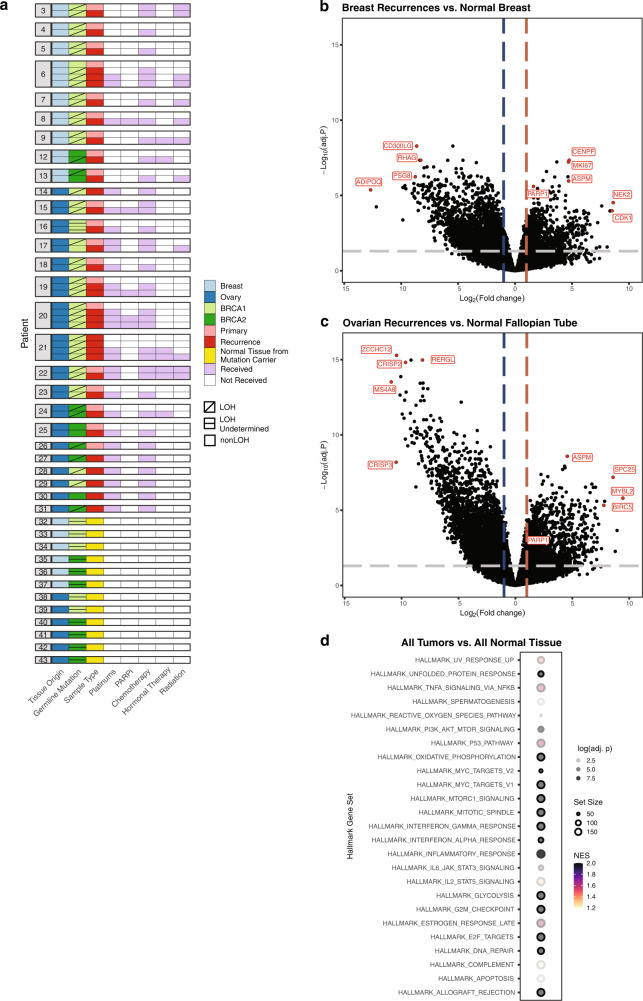
Global transcriptomic programs detected by RNA sequencing. **a** 66 samples used for RNA sequencing. Tumors are displayed in chronological order by patient, with the primary tumor at the top and latest recurrence at the bottom. Patients 28–31 had recurrent tumors only. Patients 32–43 were *BRCA1/2* mutation carriers with no prior history of cancer or cancer treatment; their samples are normal tissue from breast and fallopian tube. LOH *BRCA1/2* allele-specific loss of heterozygosity; PARPi PARP inhibitor. **b** Differential gene expression in breast tumor recurrences vs. normal breast tissue. **c** Differential gene expression in ovarian tumor recurrences vs. normal fallopian tube tissue. For **b** and **c**, a positive log_2_(fold-change) indicates genes with increased expression in recurrent tumors. Adjusted *p*-values were computed based on linear modeling of mean-variance trends (limma). **d** Hallmark Gene Sets enriched in genes with increased expression in primary and recurrent breast and ovarian tumors compared to normal breast and fallopian tube tissue (all adj. *p* < 0.05 per gene set enrichment analysis). NES normalized enrichment score.

We performed RNA sequencing (RNA-seq) on 50 primary and recurrent tumors from the WES cohort, adding in recurrences from four additional patients (subjects 28–31, Fig. 3a and Supplementary Data 1). We also conducted RNA-seq on 12 normal breast and fallopian tube samples from prophylactic surgeries in *BRCA1/2* mutation carriers (subjects 32–43, Fig. 3a; Supplementary Fig. 7a and Supplementary Data 1). We used hierarchical clustering to assess sample relatedness across the cohort (Supplementary Fig. 7b). In the resulting dendrogram, many paired primary and recurrent tumors were not each other’s closest relatives (11/19 tumor pairs). In general, ER- breast tumors clustered closer to ovarian tumors than ER+ breast tumors, which in turn clustered closest to normal samples. Sample relatedness appeared to be most informed by ER status and tissue of origin. We further assessed sample clustering using principal components (Supplementary Fig. 7c). We did not observe any clustering associated with tumor origin (breast or ovarian), tumor type (primary or recurrent), or germline mutation (*BRCA1* vs. *BRCA2*).

*PARP1* expression was significantly increased in breast and ovarian recurrences compared to normal tissue (log_2_FC = 1.62, adj. *p* = 2.52 × 10^−6^ and log_2_FC = 9.44, adj. *p* = 1.58 × 10^−6^) (Fig. 3b, c). *PARP1* expression was also increased in breast primary tumors (log_2_FC = 1.43, adj. *p* = 3.26 × 10^−5^) (Supplementary Fig. 8a). Primary and recurrent ovarian tumors both showed increased expression of *MYBL2*, for which PARP1 is a putative coactivator (Fig. 3c and Supplementary Fig. 8b)^52^. Primary and recurrent breast tumors showed increased expression of *MKI67* (Ki67), which has been positively correlated with *PARP1* expression in other breast tumor cohorts (Fig. 3b and Supplementary Fig. 8a)^53^. Overall, *PARP1* expression in all tumors exceeded the mean *PARP1* expression in normal tissue samples, an effect that did not depend on the presence of a *PARP1* gain or amplification (*p* = 3.17 × 10^−7^ and *p* = 5.01 × 10^−5^ for tumors with and without *PARP1* gain or amplification, respectively, Supplementary Fig. 9).

As the average log_2_(counts per million) for individual genes were similar between breast and ovarian tumors overall (*R*^2^ = 0.934), we combined all *BRCA1/2* mutation-associated tumors to identify the major transcriptomic differences compared to normal tissues. Using GSEA, we found enrichment of 25 Hallmark gene sets (all adj. *p* < 0.01, Fig. 3d, Supplementary Data 13). The identified gene sets included several pathways also enriched for copy number gains (Fig. 2g): IFN*α* and IFN*γ* signaling, MTORC1 signaling, response to UV light, and TNF*a* signaling via NF-*k*B. Increased expression of these gene sets was also identified in GSEA stratified by tumor type (Supplementary Fig. 8c, d and Supplementary Data 13).

### Analysis of gene fusions in primary and recurrent tumors

**Fig. 4:**
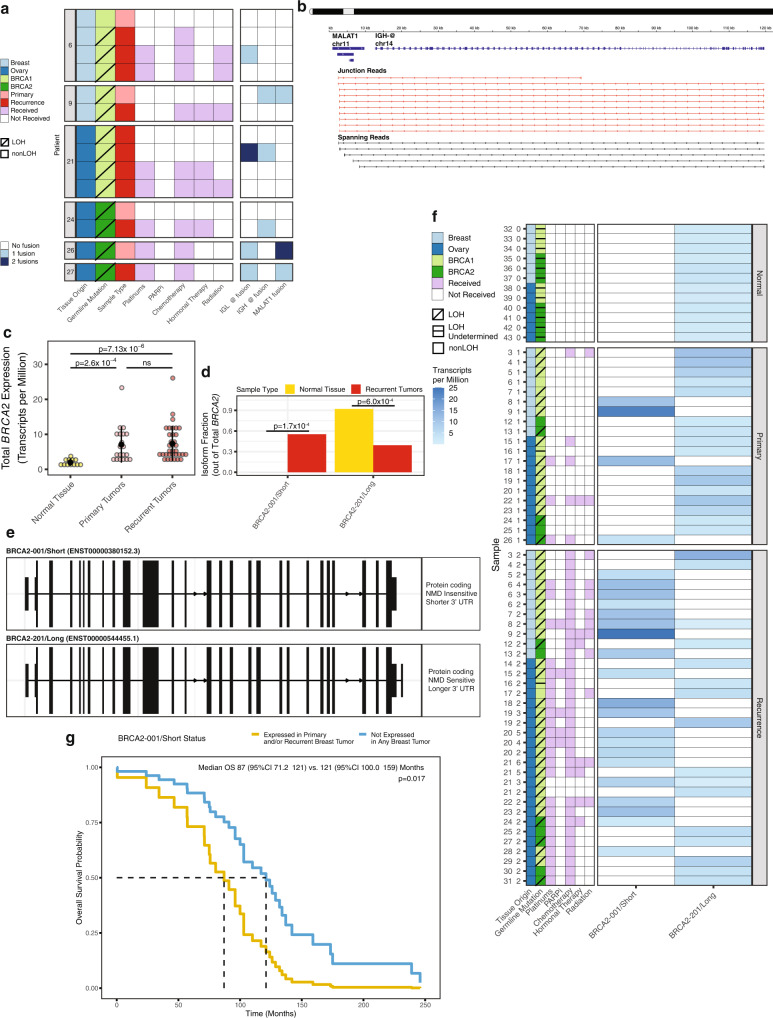
Gene fusions and isoform switching detected by RNA sequencing. **a** Clinical characteristics of patients in which *IGH-@, IGL-@*, and *MALAT1* fusions were identified. Tumors are displayed in chronological order by patient, with the primary tumor at the top and latest recurrence at the bottom. “1 fusion” refers to a translocation with one other gene. LOH *BRCA1/2* allele-specific loss of heterozygosity; PARPi PARP inhibitor. **b** Example of *MALAT1-IGH-@* gene fusion IGV tracks from patient 9. Junction reads (red, middle track) represent split RNA-seq reads used to map the fusion breakpoint. Spanning reads (black, bottom track) represent paired-end reads of fragments that span, but do not directly overlap, the fusion breakpoint. **c** Expression of total *BRCA2* (all isoforms) in recurrences vs. normal samples (*n* = 66 biologically independent samples from 39 patients). Data are expressed as mean values +/– SD. NS not significant. Adjusted *p*-values were computed based on linear modeling of mean-variance trends (limma). **d** *BRCA2* isoform usage (*BRCA2* isoform expression normalized to total *BRCA2* expression) in recurrences vs. normal samples; *q*-values computed using DEXSeq within isoformSwitchAnalyzer. **e** *BRCA2* isoforms involved in isoform switching event. NMD nonsense-mediated decay, UTR untranslated region. **f** *BRCA2* isoform expression by sample and group for entire RNA sequencing cohort. **g** Overall survival (OS) curve for patients that expressed *BRCA2-001/Short* in any (primary or recurrent) breast tumor compared to those that did not. Survival proportions and *p*-value were calculated using a Cox proportional hazards model tested for significant associations with ER status, age at diagnosis, tumor stage at diagnosis, and patient recurrent status (*α* = 0.05, see Methods).

We investigated whether gene fusions could be potential drivers in *BRCA1/2* mutation-associated tumors. We limited our analysis to previously described gene fusions with >5 junction-spanning reads (range 5–228 reads) found in ≥3 patients. Six of 54 (11%) tumors had fusions involving genes for immunoglobulin light and/or heavy chains (IGL@, IGH@) (Fig. 4a, b and Supplementary Data 14). Fusions of *MALAT1*, a lncRNA associated with poor survival in metastasis in several tumor types, were recently observed at high frequency (13/18, 72%) in ovarian cancer and TNBC patients after PARPi treatment^54,55^. We found *MALAT1* fusions in one breast and two ovarian (3/54, 5.5%) tumors (Fig. 4b). We did not identify any fusions involving efflux pump *ABCB1*, which have been previously associated with multidrug resistance^17,27^.

### Differential *BRCA2* isoform usage in primary and recurrent tumors

As mRNA isoform switching events are a well-recognized phenomenon in cancer, we evaluated differential transcript usage among primary tumors, recurrent tumors, and normal tissue samples^56^. We identified an isoform switching event between recurrences and normal tissue, involving two protein-coding *BRCA2* transcripts. Overall, *BRCA2* gene expression was significantly higher in breast and ovarian recurrent tumors compared to normal breast and fallopian tube tissue (adj. *p* = 7.13 × 10^−6^, Fig. 4c), whereas *BRCA2* was intermediately expressed in primary tumors. The increase in *BRCA2* expression was also observed in breast and ovarian recurrences when compared separately to matched normal tissue (both log_2_FC ≥ 1.79, adj. *p* < 1 × 10^−3^). We found that recurrent tumors preferentially expressed a shorter transcript (GRCh37.p13 ENST00000380152.3 or BRCA2-001, hereafter referred to as “*BRCA2-001/Short*,” *q* = 1.7 × 10^−4^). In contrast, normal samples favored a longer transcript (GRCh37.p13 ENST00000544455.1 or BRCA2-201, hereafter referred to as “*BRCA2-201/Long*,” *q* = 6.0 × 10^−4^) (Fig. 4d). No significant difference in isoform usage between primary tumors and normal tissues was observed. Both transcripts contain all the functional domains, such as the BRC repeats and OB domains. *BRCA2-201/Long* has a longer 3’ UTR, predicted to be sensitive to nonsense-mediated decay (NMD); whereas with a shorter 3’ UTR, *BRCA2-001/Short* is predicted to be NMD-insensitive, due to decreased distance between the stop codon and terminal exon junction complex (Fig. 4e)^57^. The differing region of the *BRCA2* 3’ UTR contains binding sites for RNA-binding proteins ELAVL1 (HuR) and PABPC1 (both FDR *q* < 0.01, Supplementary Fig. 10a), based on RNA immunoprecipitation sequencing (RIP-seq) experiments in human B cells (GEO Accession GSE35585, (https://www.ncbi.nlm.nih.gov/geo/query/acc.cgi?acc=GSE35585). The presence of HuR binding sites in pre-mRNA introns and 3’ UTR has been directly associated with increased mRNA stability^58^. PABPC1 has been to shown to inhibit NMD in mRNA transcripts with long 3’ UTRs^59,60^.

We observed that expression of each *BRCA2* isoform was mutually exclusive in 64 of 66 (97%) RNA-seq samples, with increasing enrichment of the short isoform from the normal to primary to recurrence groups (*p* = 0.001, Fig. 4f, Supplementary Data 15). Across all tumor samples, expression of *BRCA2-001/Short* was slightly more common in breast tumors (10/20; 50%) than in ovarian tumors (14/34; 42%). *BRCA2-001/Short* was expressed in 8/11 (73%) breast recurrences compared to 12/23 (52%) ovarian recurrences. In general, *BRCA2-001/Short* was expressed more frequently in *BRCA1* mutation-associated tumors (21/42; 50%) than in *BRCA2* mutation-associated tumors (3/12; 25%). This difference was largest within the recurrence group; *BRCA2-001/Short* was expressed in 18/27 (67%) *BRCA1* mutation-associated recurrences compared to 2/7 (29%) *BRCA2* mutation-associated recurrences, but non-significant. There were no apparent associations with treatment history or LOH status.

We confirmed expression of *BRCA2-001/Short* and *BRCA2-201/Long* in primary and recurrent tumors by quantitative reverse transcription PCR (RT-qPCR; Supplementary Fig. 10b, c and Supplementary Data 16). To further validate these results, we assessed *BRCA2* isoform expression in an independent cohort of 42 *BRCA1/2* mutation-associated primary breast and ovarian tumors (Supplementary Data 1, Supplementary Fig. 11a). As in our discovery set, primary tumors in the validation cohort more commonly expressed *BRCA2-201/Long* (33/42; 79%, Supplementary Data 15). Expression of the shorter isoform *BRCA2-001/Short* was similarly uncommon in breast vs. ovarian primary tumors (28%, 29%) and in *BRCA1* vs. *BRCA2* mutation-associated primary tumors (28%, 31%).

Next, we tested whether expression of *BRCA2-001/Short* affected overall survival across 67 *BRCA1/2* mutation carriers (from discovery and validation cohorts) for whom we had survival data and RNA sequenced from at least one tumor (Supplementary Data 15). Survival time for breast cancer patients did not have a significant association with ER status (*p* = 0.16), age at diagnosis (*p* = 0.47), *BRCA1* vs. *BRCA2* germline mutation (*p* = 0.4), tumor stage at diagnosis (*p* > 0.74), or patient recurrence status (*p* = 0.58) (Supplementary Data 15). Out of 34 breast cancer patients assessed, those with *BRCA2-001/Short* expression in any tumor had significantly worse overall survival compared to patients whose tumors did not express this alternative isoform (*p* = 0.017, HR = 2.535, 95% CI 1.179–5.45) (Fig. 4g). Median overall survival was nearly 3 years shorter for patients whose tumor(s) expressed *BRCA2-001/Short* (87 months, 95% CI 71.2–121) compared to those whose tumors did not (121 months, 95% CI 100.0–159). Breast tumors expressing *BRCA2-001/Short* were evenly split between patients that eventually recurred (54%) and those that did not (46%). Expression of this shorter *BRCA2* isoform was not correlated with overall survival in 33 ovarian cancer patients after adjustment for patient recurrence status (Supplementary Fig. 11b).

### Analysis of DNA damage response and immune checkpoint proteins

**Fig. 5:**
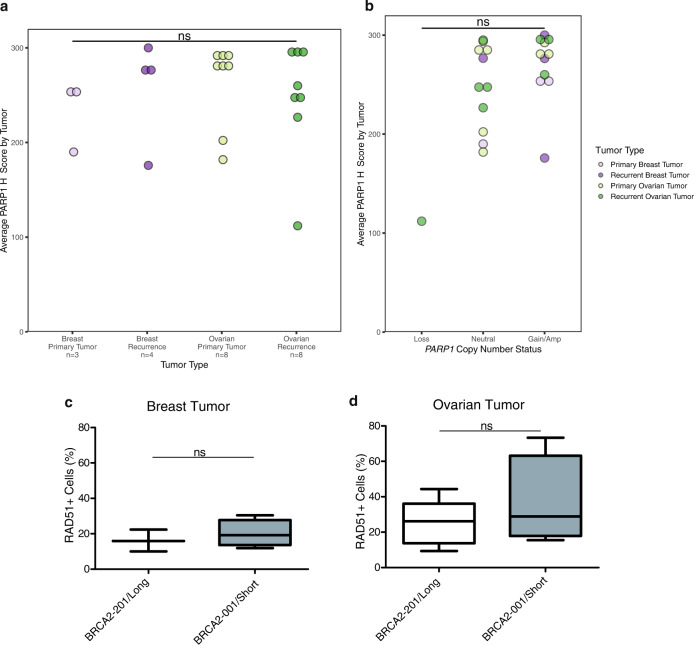
Validation of prior results by PARP1 and RAD51 protein expression. **a** PARP1 nuclear positivity (average tumor *H*-score) by tumor type, for 23 tumors in tissue microarrays. NS not significant. **b** PARP1 nuclear positivity (average tumor *H*-score) by *PARP1* copy number status across all tumors. For **a** and **b**, groupwise differences were assessed by Kruskal–Wallis test, followed by Dunn’s test with Bonferroni correction (*α* = 0.05). **c** Percent of RAD51+ cells in primary (*n* = 3) and recurrent (*n* = 4) breast tumors based on *BRCA2* transcript usage. **d** Percent of RAD51+ cells in primary (*n* = 6) and recurrent (*n* = 7) ovarian tumors based on *BRCA2* transcript usage. For **c** and **d**, boxplot elements are as follows: median, center line; box limits, first and third quartiles (spanning the IQR, interquartile range); whiskers, 1.5x IQR in each direction; outliers plotted individually. Groupwise differences in **c** and **d** were assessed by two-sided Wilcoxon rank sum test (*α* = 0.05). Tumors without RNA-seq data (*n* = 2) or expressing both *BRCA2* isoforms (*n* = 1) were excluded from this analysis.

We next assessed whether *PARP1* copy number gains and mRNA expression translated to high PARP1 protein expression. We performed immunohistochemistry (IHC) for PARP1 on three tissue microarrays (TMAs) containing 23 primary and recurrent *BRCA1/2* mutation-associated tumors from our sequencing cohort (Supplementary Fig. 12a–d, Supplementary Data 1, Supplementary Methods). PARP1 protein was highly expressed across the cohort of tumors (mean *H*-score 252.5, median *H*-score 276.4) with no significant differences by tumor type (Fig. 5a). As in the RNA-seq results, high PARP1 expression did not depend on the presence of a *PARP1* gain or amplification (Fig. 5b). However, the only tumor with a *PARP1* copy number loss had a relatively low *H*-score of 112. Overall, our results from RNA-seq and IHC suggest that PARP1 is highly expressed in *BRCA1/2* mutation-associated breast and ovarian cancers.

Next, we assessed DNA damage markers and immune checkpoint proteins using the PhenoCycler platform (Supplementary Fig. 13a–d, Supplementary Table 2, Supplementary Methods). We found no significant differences in percent of RAD51+ tumor cells between primary and recurrent tumors (Supplementary Fig. 14a, b). However, we found that the percent of (tumor and stromal) RAD51+ cells trended higher in tumors expressing *BRCA2-001/Short* compared to those expressing *BRCA2-201/Long* (Fig. 5c, d). We found no differences in percent of (tumor and stromal) CTLA-4+ or PD-1+ cells between primary and recurrent tumors (Supplementary Fig. 14c–f).

### Tumor evolution

**Fig. 6:**
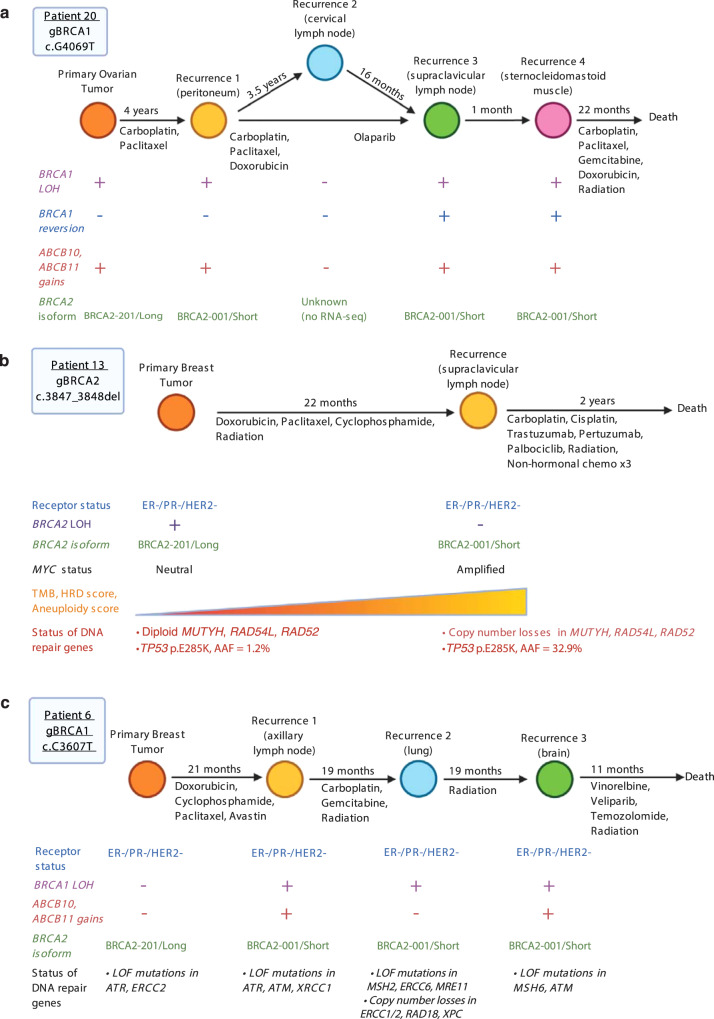
Evolution of *BRCA1/2* mutation-associated breast and ovarian tumors in three patients. **a** Genetic and clinical features in Patient 20, a *BRCA1* mutation carrier with ovarian cancer. LOH allele-specific loss of heterozygosity. **b** Genetic and clinical features in Patient 13, a *BRCA2* mutation carrier with breast cancer. AAF alternative allele fraction. **c** Genetic and clinical features in Patient 6, a *BRCA1* mutation carrier with breast cancer. For **a**–**c**, display is limited to tumors sequenced for this study. Copy number losses refer to genes with total copy number of 0 or 1; copy number gains refer to genes with total copy number ≥4. Loss of function (LoF) mutations reported here are from high-depth targeted sequencing.

We present the multi-omic results from three patients for an in-depth analysis of the relationship between primary and recurrent tumors. Patient 20 was a *BRCA1* mutation carrier with an ovarian primary tumor and four recurrences (Fig. 6a). Over 9 years during which the patient received multiple lines of therapy, the tumor maintained *BRCA1* LOH, copy number gains in *ABCB10/11*, high aneuploidy scores (≥13), *MYC* amplifications (CN ≥ 10) and *PARP1* gains (CN = 4) (Supplementary Fig. 3a). However, *BRCA2* isoform usage evolved, with her primary tumor expressing *BRCA2-201/Long* and recurrences expressing *BRCA2-001/Short*. Recurrence #2 had different genomic features (apparent nonLOH and a completely unique set of driver mutations, including a *PARP1* mutation), which suggest evolution from a distinct clone (Supplementary Fig 3b and Supplementary Data 2). The patient eventually progressed on PARPi, and a *BRCA1* reversion was identified (Supplementary Fig. 15a, b).

Patient 13 was a *BRCA2* mutation carrier with TNBC. Her LOH primary yielded a nonLOH recurrence (Fig. 6b). This recurrent tumor was the only one that underwent LOH reversal without a concomitant decrease in HRD score (Fig. 2b, left panel). The primary tumor had a low-level pathogenic *TP53* mutation with AAF of 1.2%, which increased to 32.9% in the recurrence, suggesting the outgrowth of a nonLOH *TP53*-mutant subclone (Supplementary Data 3). Although the recurrence shared multiple variants with the primary, multiple additional mutations emerged, reflective of an increase in TMB (2.1 to 12.8, Supplementary Data 4). The tumor also showed *BRCA2* isoform switching and a *MYC* amplification. The recurrence had copy number losses and mutations in HR and other DNA damage response genes, including *MUTYH, RAD54L*, and *RAD52*. RAD52 specifically is required for survival of BRCA2-deficient cells^61^. Aneuploidy and HRD scores all increased upon recurrence, indicating continued accumulation of genomic instability and mutations.

For Patient 6, a *BRCA1* mutation carrier, we evaluated one primary tumor and three recurrences of TNBC (Fig. 6c). The recurrent tumor had allele-specific LOH and a *BRCA2* isoform switch (Supplementary Fig. 2b). Two recurrences had copy number gains in *ABCB10/11*. Over the course of tumor progression, recurrences underwent loss of DNA damage response and repair genes (by copy number loss or LoF mutation). These deleterious alterations varied across recurrences but clustered into the same pathways.

## Discussion

In this study, we evaluated multi-omic features of paired primary and recurrent tumors from 27 *BRCA1/2* mutation carriers, representing 13 breast cancers (primarily TNBC) and 14 ovarian cancers (mostly high-grade serous). Patients received a ‘real world’ mix of chemotherapy and radiation, in addition to platinums and PARPi, prior to collected recurrences. We assessed HRD, aneuploidy, somatic mutations, copy number variation, and global transcription in paired *BRCA1/2* mutation-associated breast and ovarian tumors, focusing on features associated with recurrence.

We initially assessed LOH status in matched primary and recurrent tumors to determine whether *BRCA1/2* deficiency varied over the course of tumor evolution. Seven pairs of primary and recurrent tumors demonstrated changes in allele-specific LOH status, more commonly in breast cancer. We observed the highest frequency of nonLOH tumors (50% of tumors, 100% of patients) in *BRCA2* mutation-associated breast cancers, as has been previously reported^48^. We also found that LOH discordance was more common in patients with ER+ breast cancer and those without somatic *TP53* LoF mutations. Three breast cancers (one *BRCA1*, two *BRCA2*) developed allele-specific LOH upon recurrence, associated with a significant increase in HRD score. Each primary/recurrent pair contained shared variants with relatively stable AAFs, demonstrating they arose from the same clone. However, the recurrences with LOH had multiple additional somatic variants, which were shared between recurrences (if multiple were collected). Thus, the development of allele-specific LOH appeared to be part of tumor progression in these cases. These data are consistent with the higher rates of *BRCA1/2* allele-specific LOH reported for patients enrolled in clinical trials with metastatic breast cancer compared to those with primary tumors^48,62^.

Interestingly, two breast (both *BRCA2*) and two ovarian (both *BRCA1*) tumors underwent an apparent reversal of allele-specific LOH, reflected by lower HRD scores in nonLOH recurrences. We postulate that in some cases, recurrences are associated with the outgrowth of a nonLOH clone secondary to therapeutic pressures, which is supported by the trajectory of genomic changes in patients 10 and 13. For patient 10, one recurrence (10-2) does not demonstrate LOH and has a lower HRD score. In contrast, the primary and second recurrence (10-3) do have LOH, with the primary tumor also demonstrating Signature 3. 10-1 and 10-3 share several variants at high levels not seen in 10-2, although other variants are shared amongst the primary and both recurrences. Similarly, the recurrence in patient 13 demonstrates the same *TP53* mutation as the primary, detected with a much higher AAF. Our observations of outgrowth of sub-clones without LOH are consistent with the findings of Imyantiov and colleagues, who found that neoadjuvant therapy with platinums resulted in rapid selection of pre-existing BRCA1-proficient cells, so that the tumors post-treatment did not exhibit *BRCA1* LOH^31,63^. Our data extend that finding, suggesting that LOH reversal can also occur in tumors treated with non-platinum chemotherapy and radiation. Of note, BRCA1 proficiency appeared to be disadvantageous to tumors in the absence of platinum exposure, as tumor relapses usually re-acquired *BRCA1* LOH during therapy holidays, an effect we could not evaluate in this cohort^31,63^.

The etiology for the other tumors demonstrating LOH reversal is not as straightforward to characterize. It is possible for some individuals, differences between primary and recurrent tumors could be due to the existence of a second unrelated primary tumor, not clinically appreciated. Although LOH status was consistent across tumors in patient 21, the pattern of variants suggests this phenomenon. The primary tumor and two recurrences (ovary and intra-abdominal) had the same *TP53* mutation and are clearly closely related with multiple overlapping variants. In contrast, three of the recurrences (breast, lymph node) share a different *TP53* mutation and no additional pathogenic variants, suggesting the breast tumor was in fact a second primary. For ovarian cancer patients 17 and 20, nonLOH recurrences both emerged post-platinums and did not share pathogenic variants with the primary and other recurrences. Both tumors had high purity and were included in high-depth targeted sequencing, so variants were unlikely to be missed. In these cases, it is difficult to know if nonLOH recurrences arose from a subclone or were associated with a distinct unknown primary tumor. Thus, multiple etiologies may underlie the observation of allele-specific LOH reversal, supporting genomic analysis of recurrences to ensure optimal therapeutic selection in this patient population.

We also evaluated multi-omic data to identify changes associated with recurrence across tumors. HRD and aneuploidy scores did not change significantly with recurrence, consistent with prior findings that HRD scores do not increase upon post-platinum recurrence in *BRCA1/2* mutation-associated ovarian cancer^29^. We also did not observe significant increases in TMB. Although a prior observation suggested that TMB increases upon recurrence post-platinum therapy in *BRCA1/2* mutation-associated ovarian cancers, the study included only five primary/recurrent ovarian tumor pairs^17^. Consistent with prior studies of ovarian cancers, our results suggest that *BRCA1/2* mutation-associated malignancies accrue most of their genomic scarring and mutations early in tumorigenesis, and increasing genomic instability is not a major factor driving recurrence.

We identified several significant differences between primary and recurrent tumors with respect to somatic mutations, copy number variation, and gene expression. We identified a *TP53BP1* mutation following PARP inhibition in a *BRCA1* mutation carrier, in line with prior studies demonstrating that *TP53BP1* loss mediates PARPi resistance in *BRCA1-*mutant cells and murine mammary tumors^18,19^. Several cancer-associated gene sets were exclusively enriched for LoF mutations within breast and ovarian recurrences, including those for apoptosis; glycolysis; and P53, TGF*β*, Notch, and MTORC1 signaling. Hedgehog signaling genes accumulated both LoF variants and copy number deletions exclusively within recurrences, suggesting that Hedgehog signaling is required for primary tumorigenesis (as in sporadic breast and ovarian cancers) but dispensable or dysregulated in recurrence^64,65^. Breast and ovarian recurrences had distinct copy number profiles, despite having similar HRD and aneuploidy scores. Notably, recurrences of both type had *ABCB10* amplifications, suggesting a shared mechanism of chemoresistance only observed in vitro previously^50^. In a subtractive copy number analysis, we also observed recurrence-specific deletions of *KMT2C* and *MAD1L1*, and frequent mutations across the cohort in *KMT2C*. Loss of *KMT2C (MLL3)* is associated with progression and metastatic disease in multiple cancer types, including *BRCA1/2* mutated breast and ovarian cancers^66–70^. Mitotic arrest deficient-like 1 (MAD1L1) is a component of the mitotic spindle-assembly complex and repressor of TERT; its loss increases chromosomal instability and could allow telomere lengthening^71,72^. Although the role of *MAD1L1* in cancer has not been fully elucidated, some studies suggest loss of expression is associated with metastatic disease and poor prognosis^73,74^. Our results suggest that *MAD1L1* is a tumor suppressor in the context of *BRCA1/2* mutation-associated tumors, loss of which is associated with recurrent disease. Ultimately, the genetic and transcriptomic features identified as unique to recurrences could represent drivers and therapeutic targets for late-stage *BRCA1/2* mutation-associated tumors.

We initially observed that *PARP1* amplifications were statistically significant (90% CI per GISTIC) in recurrent tumors and observed most frequently (78%) in breast cancer recurrences. We then found that *PARP1* was gained or amplified across all tumor groups. We identified up to 8-fold amplifications centering on a minimal common region of chr1:226,474,131-227,148,258, which fully encompasses only three other genes (*ITPKB*, *PSEN2, STUM*) in addition to *PARP1*. In our and the TCGA cohorts of primary tumors, we found a similar frequency of *PARP1* gains and amplifications across g*BRCA1/2* and sporadic HR-WT breast and ovarian tumors. Variation in methods likely accounts for the different prevalence of *PARP1* amplification reported in cBioPortal for TCGA PanCancer ovarian (5% vs. 22%) and breast (9% vs. 25%) cohorts^75^. We then observed (over two-fold) increases for *PARP1* expression in primary and recurrent breast tumors, as well as ovarian recurrences, independent of *PARP1* copy number. The correlation of *PARP1* copy number with *PARP1* expression approached significance, suggesting that with a larger sample set we might find an association, as has been previously observed^76^. Our results also agree with prior reports of high *PARP1* expression in unselected ovarian and hormone receptor-negative breast cancers^77–79^. Increased nuclear PARP1 protein has been correlated with decreased relapse-free and overall survival in breast cancer and AML^80,81^. In this study, we present evidence of *PARP1* overexpression as a near-ubiquitous feature of both primary and recurrent *BRCA1/2* mutation-associated tumors, corroborated at the protein level. Our study supports that *PARP1* copy number gains represent a common (but not exclusive) route to *PARP1* overexpression, and that such gains are common in breast and ovarian tumors regardless of *BRCA1/2* status^76^. A highly positive correlation between PARP1 expression and PARPi resistance previously has been demonstrated in breast cancer cell lines, wherein the *BRCA1*-mutated cell line HCC1937 had both the highest level of PARP1 expression and greatest resistance to PARPi^82^. Similarly, increased PARP1 expression has been correlated with PARPi resistance in AML cell lines^83^. Increased levels of PARP1 protein may stoichiometrically dilute PARPi, which trap PARP1/2 complexes on DNA for synthetic lethality^84,85^. Thus, levels of PARP1 protein, which could be increased due to amplifications or overexpression, should be explored as biomarkers of therapeutic response or clinical outcomes in recurrences from patients treated with PARPi^86–89^.

We assessed transcriptomic differences in primary and recurrent tumors and found two different isoforms of *BRCA2*. In silico predictions suggest that *BRCA2-001/Short* and *BRCA2-201/Long* differ in regulation of mRNA stability and/or localization, as dictated by NMD sensitivity and regulation by RNA-binding proteins^56,57^. Expression of *BRCA2-001/Short* was significantly more common in recurrent tumors and further enriched among breast tumors and *BRCA1* mutation carriers. We hypothesize that the isoform switch may represent a mechanism by which *BRCA1* mutation-associated tumors can modulate *BRCA2* transcript stability to retain HR function and enhance tumor survival. In nonLOH *BRCA2* mutation-associated tumors, expression of the shorter isoform could also protect mRNA expression from the wild-type *BRCA2* allele. Our survival analysis offers support for a correlation between *BRCA2* isoform expression and clinical outcomes. We found that breast cancer patients lacking *BRCA2-001/Short* expression in any tumor (primary or recurrences) had significantly improved overall survival (median 87 vs. 121 months), suggesting it could be a prognostic biomarker. We did not observe differences in survival based on isoform usage in ovarian cancer patients. In existing literature, multiple mechanisms have been shown to restore BRCA2 function in platinum and PARPi resistance, and isoform switching could represent an additional mechanism^25,26,90–92^. This hypothesis is also supported by increased RAD51 positivity (a readout of BRCA2 activity) among tumors expressing *BRCA2-001/Short*. Taken together, our results suggest that *BRCA1* mutation-associated tumors, as well as nonLOH *BRCA2* mutation-associated tumors, could benefit from enhanced stability of *BRCA2* mRNA. We therefore propose that *BRCA2* isoform switching represents a tumorigenic driver in *BRCA1/2* mutation-associated cancers.

Although this cohort represents a relatively large set of paired primary and recurrent *BRCA1/2* mutation-associated tumors, the number of matched primary and recurrent tumors was still relatively small. As a result, we were limited in our ability to identify correlations between genomic or transcriptomic features and treatment, which was quite heterogeneous. The cohort includes few post-PARPi recurrences, as PARPi use was relatively rare at the time of sample collection. We focused our efforts on identifying similarities and differences between paired tumors across the cohort. Seven of the 27 patients had multiple recurrences. We accounted for this enrichment in our GSEA analyses, by counting a gene as mutated or gained/lost only once even if observed across common recurrences. We also limited pairwise statistical comparisons to one recurrence per patient, selected at random. However, for MutSigCV and GISTIC analyses, all recurrences were included to improve our power to detect recurrence-specific events.

Since we employed bulk sequencing techniques, we may not fully appreciate tumor heterogeneity associated with recurrent disease. We attempted to use the same formalin-fixed paraffin-embedded (FFPE) block and samples for multi-omic sequencing, but it was not always possible due to sample availability. For the same reason, we could not assess *BRCA1* promoter methylation, another possible route to biallelic *BRCA1* loss in *BRCA1* mutation-associated tumors. However, we did confirm *BRCA1* expression in all nonLOH *BRCA1* mutation-associated tumors with RNA-seq. Additionally, *BRCA1* promoter methylation does not always abrogate *BRCA1* expression^93–95^. Further validation of key results (LOH transitions, *PARP1* amplifications, *BRCA2* isoform switching) in an independent cohort of paired primary and recurrent *BRCA1/2* mutation-associated breast and ovarian tumors will be important. Although we were able to determine that the *BRCA2* isoform switching event was associated with decreased survival in two independent sample sets, additional cohorts are needed to confirm, and mechanistic studies remain to be done to confirm functionality.

Ultimately, these results suggest key biological features of therapy-resistant recurrences, thereby highlighting therapeutic possibilities for *BRCA1/2* mutation carriers with cancer. We identified allele-specific LOH transitions in 25% of patients, underlining the plasticity of *BRCA1/2* status after treatment with DNA damaging agents. These results suggest that sequencing the most recent tumor or circulating DNA will be most informative in planning personalized treatment. Lastly, we found that *BRCA2* isoform switching may be a pro-tumorigenic event that appears in both breast and ovarian recurrences and is associated with reduced breast cancer patient survival. Further studies are needed to assess whether these mechanisms are specific to *BRCA1/2* mutation-associated breast and ovarian cancers, or also observed in the context of other tumor types or other DNA repair defects.

## Methods

### Acquisition of tumor, germline, and normal tissue specimens from *BRCA1/2* mutation carriers

Patients gave written informed consent for research use of germline DNA, tumor specimens, and clinical data—including the publication of patient age –under IRB approved protocol at the University of Pennsylvania and Vall d’Hebron. Participants were not compensated. Eligible patients met the following criteria: (1) diagnosis of breast or ovarian cancer; (2) positive genetic test result for pathogenic germline mutation in *BRCA1* or *BRCA2* from a Clinical Laboratory Improvement Amendments (CLIA)-approved laboratory; (3) available archived germline DNA from blood or saliva; (4) available formalin-fixed paraffin-embedded (FFPE) blocks from the primary tumor; and (5) available FFPE blocks from at least one recurrence matching the primary tumor. Matched recurrent tumor samples were identified using manual review of patient charts and pathology reports, supervised by KNM and SMD. All clinical phenotypic data were collected through manual chart review. Patients 1-27 met all study criteria and comprised the primary/recurrent tumor cohort. An additional four patients (Patients 28–31) lacked an available primary tumor sample (meeting all but criterion 4) and were included only in groupwise RNA sequencing analyses.

We also identified 12 *BRCA1/2* mutation carriers as a control group for RNA sequencing analyses. Eligible patients met the following criteria: (1) positive genetic test result for pathogenic germline mutation in *BRCA1* or *BRCA2* from a CLIA-approved laboratory; (2) no prior history of cancer or cancer treatment; and (3) available FFPE blocks from prophylactic mastectomy or salpingo-oophorectomy surgeries. We extracted and sequenced RNA from normal breast and fallopian tube samples from these patients. Of note, fallopian tube samples were used as controls for ovarian cancer, for which the tissue of origin is the fallopian tube^96^.

### Sample acquisition of primary tumor validation cohorts from TCGA and Penn

For validation of *PARP1* copy number gains, we used WES from primary breast and ovarian tumors in TCGA cohorts (Supplementary Data 12). Level 1 WES binary alignment map (bam) files from TCGA Breast and Ovarian cohorts were obtained from Genomic Data Commons (https://gdc.cancer.gov/)^48,51^. Tumors were excluded from analysis for any of the following reasons: (1) failed quality control; (2) somatic *BRCA1/2* loss as indicated by mutation, low expression, or *BRCA1* promoter methylation; or (3) germline or somatic mutations in genes related to homologous recombination (see Supplementary Data 12). TCGA tumors were determined to be associated with *BRCA1* or *BRCA2* pathogenic variants if they had the following: (1) pathogenic *BRCA1/2* variants (either known missense or loss of function, both known and unknown); (2) germline variant allele frequency exceeding 30%; and (3) total allele depth exceeding 30 reads in both germline and tumor samples^48,51^. Tumors were grouped in g*BRCA1/2* or Homologous Recombination-Wild Type (HR-WT) groups, as visualized in Supplementary Fig. 6a.

For validation of *BRCA2* isoform switching, we evaluated an independent cohort of 42 primary breast and ovarian tumors from patients with *BRCA1/2* mutations, also consented under the University of Pennsylvania approved IRB protocol. Eligible patients had a diagnosis of breast or ovarian cancer with available FFPE tumor tissue and positive genetic test result for pathogenic germline mutation in *BRCA1* or *BRCA2* from a CLIA-approved laboratory. RNA extraction, sequencing, and analyses were identical to those used for primary/recurrent cohort samples.

### Pathological review of FFPE specimens

FFPE tumors were collated and sectioned by the Tumor Tissue and Biospecimen Bank at the University of Pennsylvania, then stained using hematoxylin and eosin. For tumor samples, staining was reviewed and marked by AN to identify sections with ≥70% invasive tumor. Normal tissue (RNA sequencing controls) was reviewed for no evidence of tumor. DNA and RNA were extracted from FFPE sections or rolls of 5–10 μm thickness. RNA extractions were performed within 1 week of sectioning to minimize degradation.

### Whole-exome and targeted sequencing of tumor and germline DNA

DNA was extracted from FFPE using standard laboratory deparaffinization, proteinase K digestion, and ethanol precipitation. Germline DNA was extracted from whole blood or saliva. Germline DNA was extracted from whole blood using sucrose-based lysis of erythrocytes, followed by proteinase K digestion and ethanol precipitation from leukocytes. Germline DNA was extracted from saliva samples using Oragene kits (DNA Genotek).

All DNA was sheared for library preparation using a Covaris sonicator. Tumor DNA libraries were prepared using the NEBNext FFPE Repair mix and NEBNext Ultra II DNA library prep kit (New England Biolabs), per manufacturer’s instructions. Germline DNA libraries were prepared using the NEBNext Ultra DNA library prep kit (New England Biolabs), per manufacturer’s instructions. DNA libraries were pooled and hybridized using SureSelect Target Enrichment System for Illumina Multiplex Sequencing (Agilent) and associated protocols. For WES, tumor and germline libraries were hybridized to SureSelect All Exon v5, SureSelect All Exon v6+COSMIC, and SureSelect All Exon v7 captures (Agilent). For targeted sequencing, tumor and germline libraries were hybridized to a custom capture, which largely utilized baits from the SureSelect All Exon platform (Agilent; see Supplementary Table 1 for gene list). DNA samples, libraries and hybridization pools were quantified using a Qubit (ThermoFisher) and fragment size was determined using a BioAnalyzer 2100 (Agilent). WES was performed using an Illumina HiSeq 4000 and targeted sequencing was performed using an Illumina NovaSeq 6000. All sequencing was performed with 150 paired-end reads by the University of Pennsylvania Next Generation Sequencing Core.

### RNA sequencing of tumor and normal RNA

Tumor RNA was extracted from the same FFPE tumor blocks from which DNA was extracted. Tumor and normal RNA samples were extracted from FFPE with the RNeasy FFPE kit (Qiagen). To preserve RNA integrity, RNA samples were extracted within 1 week of sectioning and stored at −80 °C. We used the TruSeq RNA Exome platform (Illumina) for library preparation and hybridization capture, per manufacturer protocols. Sequencing was performed with 150 paired-end reads using an Illumina HiSeq 4000 at the University of Pennsylvania Next Generation Sequencing Core.

### Bioinformatic analysis of DNA and RNA sequencing

Fastq files from whole-exome and targeted sequencing were aligned to the hg19 build of the human genome using the Burrows-Wheeler Aligner (BWA v.0.7.17-r1188)^97^. Various bam file processing operations were performed using Samtools/htslib/bcftools v1.11. The resulting bam files were processed according to Genome Analysis Toolkit (GATK v3.7) best practices (picardtools v2.20.7)^98^. WES achieved a mean depth of 98× in tumor samples (median depth 87×) and 93× in germline samples (median depth 94×). Targeted sequencing achieved a mean depth of 332× in tumor samples (median depth 304×) and 317× in germline samples (median depth 316×).

Fastq files from RNA sequencing were aligned using STAR aligner (v2.7.2a) and gene annotations from the GENCODE Human Release 19 reference assembly (https://www.gencodegenes.org/human/release_19.html)^99^. RNA sequencing achieved a mean depth of 67.6 million reads (median 55.6 million reads) for tumor and normal samples. Alignment and quantification of transcripts was performed using StringTie (v2.1.3b) to generate abundance files in Ballgown readable format^100^. We used Tximport (v1.16.1) to quantify transcript abundance from t_data.ctab files, generating “lengthScaledTPM” counts by gene^101^. Next, we used EdgeR (v3.30.3) to generate, filter, and normalize counts per million (cpm)^102^. Briefly, gene-level transcripts per million were used to create three separate DGELists: one for all samples, one for ovarian tumor and normal fallopian tube samples, and one for breast tumor and normal breast samples. All DGELists were filtered to include only genes for which transcripts were detected in ≥2 or ≥3 samples, depending on the size of the smallest biological group. Filtered DGELists were then normalized using the trimmed mean of *M* values (TMM) method^103^. Filtered, normalized DGELists were used to generate filtered, normalized cpm for each of the three comparisons. We assessed the distribution of filtered, normalized cpm across the cohort to identify any outlying samples (Supplementary Fig. 7a). Filtered, normalized cpm were used for all cohort-level and tumor-specific RNA-seq analyses, except for detection of isoform switching.

### Identification, filtering, and analysis of somatic variants

We used a union of MuTect2 (v4.1.8.1), Strelka2 (v2.9.2), VarDictJava (v1.5.1), and VarScan2 (2.4.4) to call somatic variants from whole-exome and targeted sequencing of matched tumor and germline DNA^104–107^. VarDictJava and VarScan2 were used to call and filter germline variants^104,107^. Each patient’s germline variant calls were checked to ensure detection of the same pathogenic *BRCA1/2* variant reported in their genetic test results. All variants were annotated with ANNOVAR (October 2019 release)^108^. We limited our analysis of mutational signatures to variants called by MuTect2 and with alternative allele depth of ≥10 reads. Mutational signatures were assessed separately for breast and ovarian tumor cohorts using deconstructSigs (v1.8)^44,109^.

Apart from mutational signatures, all downstream analyses of whole-exome sequencing were restricted to exonic variants that were called by and passed filtering for at least one caller and had alternative allele read depth of ≥five reads. In order to exclude common SNPs from further analyses, we also removed somatic variants with frequency in any population (PopFreqMax) ≥0.01^108^. Tumor mutational burden (TMB) was calculated for each tumor using the following equation: ${{{{{{\mathrm{TMB}}}}}}}=\frac{({n\; {{{{{\mathrm{somatic}}}}}}},{{{{{{\mathrm{exonic}}}}}}\; {{{{{\mathrm{nonsynonymous}}}}}}\; {{{{{\mathrm{SNVs}}}}}}}+{{{{{{\mathrm{indels}}}}}}})}{{{{{{{\mathrm{size}}}}}}\; {{{{{\mathrm{of}}}}}}\; {{{{{\mathrm{WES}}}}}}\; {{{{{\mathrm{capture}}}}}}}({{{{{{\mathrm{Mbp}}}}}}})}$^110^. Using the filtered variant set, we identified significantly mutated genes within primary and recurrent tumor groups using MutSigCV (v1.3)^46^.

Next, we identified gain of function (GoF) and loss of function (LoF) mutations in individual genes. LoF mutations were identified as those that met one of the following criteria: (1) frameshift mutation, (2) nonsense mutation, or (3) nonsynonymous SNV predicted to be pathogenic by REVEL score >0.5^111^. GoF mutations were defined as those that met all of the following criteria: (1) not a LOF mutation; (2) documentation in Catalog of Somatic Mutations in Cancer (COSMIC v84, https://cancer.sanger.ac.uk/cosmic); (3) occurring in a Tier 1 oncogene as defined by the Cancer Gene Census (CGC, https://cancer.sanger.ac.uk/census); and (4) matching the mutation type determined to be oncogenic for that gene by CGC. Supplementary Data 2 includes all LoF variants by sequencing type, regardless of alternative allele fraction.

Pathway analysis was performed on LoF variants using Gene Set Enrichment Analysis (GSEA) Preranked (v4.0.2 for Windows)^45^. Briefly, genes were ranked based on frequency of LoF mutations in primary and recurrent tumor groups separately. A gene could be counted as mutated only once per patient within each tumor group. This approach was used to prevent skewing of results by patients with multiple recurrences or by individual tumors accruing distinct LOF mutations in the same gene. Ranked gene lists were used as input for GSEA Preranked with Hallmark gene sets (1000 permutations). Gene set enrichment was considered significant using a cutoff of FDR < 0.25.

### Genome-wide and gene-level analysis of copy number variation

We performed allele-specific copy number analysis using Sequenza (v3.0), to identify segments of copy number variation (CNVs), as well as ploidy and cellularity estimates^47^. Sequenza-derived segments were used to calculate the components of homologous recombination deficiency (HRD): non-telomeric allelic imbalance (NtAI), large scale state transitions (LST), and genomic loss of heterozygosity (LOH). The same segments were used to calculate aneuploidy scores. Calculations were performed using custom R-scripts (https://github.com/maxwell-lab/HRDex)^48,51^.

We used GISTIC2.0 (v2.0.23, Gene GISTIC mode, with default cutoffs) to identify significant CNVs at 90% confidence in tumor cohorts^49^. Genes were highlighted from segments with residual *q* ≤ 0.05. BEDtools (v2.29.2) was used to compute Jaccard statistics for similarity between CNV sets, limited to segments of ≥50% reciprocal overlap^112^. BEDtools intersect was used to identify segments of 50% overlap between significant (90% CI, FDR *q* < 0.05) GISTIC segments in primary and recurrent tumor groups. Segments with ≥50% reciprocal overlap were considered “shared” for the purposes of this analysis, while those with <50% reciprocal overlap were considered “private” to one group. This subtractive analysis was performed separately for both amplifications and deletions, and Jaccard statistics were also computed. Primary-private, recurrence-private, and shared (intersecting) segments were annotated with RefSeq (GRCh37) genes using AnnotSV (v2.3)^113^. Genes were considered to be deleted if >50% of the locus was encompassed by a deletion. Conversely, genes were only considered to be amplified if 100% of the locus was encompassed by an amplification.

We also assessed genes within Sequenza-derived CNVs. Segments were binned based on total (integer) copy number using the following convention: CN = 0, Deletion; CN = 1, Loss; CN = 2–3, Neutral; CN = 4–5, Gain; CN ≥ 6, Amplification. Binned segments were annotated with RefSeq (GRCh37) genes using AnnotSV (v2.3) following the convention described above^113^. In the case of a gene spanning several different segments of CNV, the copy number of the gene was reported as the minimal copy number of the entire locus. For pathway analysis of genes subject to copy number gains, genes were ranked based on frequency of their presence in a segment of gain or amplification, within primary and recurrent tumor groups. Similar to our GSEA Preranked analysis of variants (see above), a gene could only be counted as gained once per patient per tumor group. We then repeated this process to rank genes again based on frequency of copy number loss or deletion by tumor group. Ranked gene lists of “gained” genes and “lost” genes were used as input for GSEA Preranked with Hallmark gene sets (1000 permutations)^45^. Gene set enrichment was considered significant using a cutoff of FDR < 0.25.

### Detection of *BRCA1/2* biallelic loss

We first checked whether *BRCA1/2* biallelic loss occurred via secondary somatic mutations as detected by whole-exome or targeted sequencing (sequencing metrics by *BRCA1/2* exon for each method are compiled in Supplementary Data 7). Specifically, we determined whether LoF mutations in *BRCA1/2* were present at an alternative allele fraction of ≥0.25. This cutoff reflected whether pathogenic secondary mutations were present in enough of the tumor to facilitate loss of the wild-type *BRCA1/2* allele. Next, we assessed loss of the wild-type *BRCA1* or *BRCA2* allele via allele-specific copy number variation from Sequenza. Briefly, we used tumor and germline bam files from WES for Sequenza input. Zygosity was estimated for all segments of somatic copy number variation based on the number of A (major) and B (minor) alleles detected. Segments with zero B alleles (only A alleles present) were deemed to have undergone LOH. Conversely, if at least one B allele was detected, a segment was considered nonLOH (heterozygous). Tumors were deemed to have undergone loss of heterozygosity (LOH) if the segment containing their pathogenic *BRCA1/2* germline mutation had LOH. When possible, we confirmed proficient *BRCA1/2* expression in nonLOH tumors with RNA sequencing. For patients with LOH transitions, we assessed the allele fraction of all *BRCA1/2* germline variants (SNPs and the pathogenic variant) across tumors to further interrogate LOH calls.

### Differential gene expression and gene fusion analysis

Filtered, normalized log_2_cpm were used to assess sample relatedness. We performed hierarchical clustering (maximum distance, average agglomeration) to generate dendrograms. Dendrograms and principal components analysis (PCA) plots were generated using the stats package in base R (v4.0.2). Between-group fold changes were computed as the difference in average log_2_cpm for a given gene between groups. Using log_2_cpm fold changes as input, we ran GSEA with Hallmark gene sets using clusterProfiler (v3.16.1) and msigdbr (v7.2.1).

We next used the limma R package (v3.44.3) to model mean-variance separately for all-tumor, ovarian-only, and breast-only DGELists. Bayesian statistics were extracted from the resulting linear models to generate adjusted *p*-values (Benjamini–Hochberg method) and log_2_(fold-change) by gene for each comparison. Significantly altered genes were extracted using decideTests in limma (global method, adj. *p* < 0.05, |log_2_(fold-change)| >1).

Gene fusions were identified using STAR-Fusion (v1.7.0) and plots were generated using FusionInspector (v2.1.0)^114^. We limited our analysis of gene fusions to those with ≥5 junction-spanning reads.

### Assessment of differential transcript usage

Abundance was re-quantified from bam files per recommendations in the Stringtie manual for novel isoform detection (http://ccb.jhu.edu/software/stringtie/index.shtml?t=manual). Briefly, starting from initial bam files, we ran Stringtie in merged mode (using GENCODE Human Release 19 as reference) to generate a merged gene transfer format (gtf) file for the cohort. We quantified abundance from initial bams using Stringtie in Ballgown mode and the merged gtf file as reference. Differential transcript usage was assessed, and switch plots generated, using IsoformSwitchAnalyzer (v1.10.0). The validation cohort of primary tumors was analyzed for isoform switching in an independent use of this workflow. RIP-seq plots were generated using UCSC Genome Browser (hg19 assembly) with the ENCODE RNA-Binding Proteins track corresponding to GEO Accession GSE35585. The track pictured for ELAVL1/HuR in Supplementary Fig. 10a shows a peak with coordinates chr13:32,973,328-32,973,692 (average enrichment 20.30, FDR *q* = 4.2 × 10^−3^). The track pictured for PABPC1 in Supplementary Fig. 10a shows a peak with coordinates chr13:32,973,450-32,973,693 (average enrichment 31.59, FDR *q* = 3.7 × 10^−3^).

### Analysis of tissue microarray (TMA) using immunohistochemistry and co-detection by indexing (CODEX)

The methods for TMA construction, immunohistochemistry of PARP1, imaging and analysis of the CODEX, including cell segmentation and marker quantification are detailed in the Supplementary Methods.

### Statistical analysis

All paired comparisons of continuous variables (HRD scores, aneuploidy scores, TMB) were tested for significance with two-sided Wilcoxon signed rank tests (*α* = 0.05). For patients with multiple recurrences, one recurrence was chosen at random for pairwise comparisons (such that *n* = 27 for each group). In the primary/recurrent cohort, groupwise differences in average *PARP1* copy number, *PARP1* mRNA expression, PARP1 H-score, and RT-qPCR ΔΔCT were assessed by Kruskal–Wallis test, with any significant results further assessed by Dunn’s test with Bonferroni correction (*α* = 0.05). We also assessed the presence of a linear relationship between *PARP1* expression and (continuous) *PARP1* copy number by Spearman’s test (*α* = 0.05). Groupwise differences in CODEX datasets were assessed by two-sided Wilcoxon rank sum test (*α* = 0.05). In TCGA cohorts, groupwise differences in average *PARP1* copy number were assessed by two-sided *t*-tests (*α* = 0.05). *PARP1* copy number is reported for primary/recurrent and TCGA tumors in Supplementary Data 11.

The adjusted *p*-value for total *BRCA2* expression was generated by linear modeling with limma (see above). The adjusted *p*-values for *BRCA2* isoform usage were computed using DEXSeq within isoformSwitchAnalyzer. Association between sample type and *BRCA2* isoform usage was determined by Mantel-Haenszel chi-squared test with continuity correction (*α* = 0.05, 1df).

Overall survival was defined as time from diagnosis to death or last follow-up. Survival metrics were calculated based on manual chart review of the electronic health record for each patient. The Penn Medicine electronic health record is indexed routinely against the National Death Index, with a time delay of ~1 year. The survival analysis was limited to 67 patients for whom we collected survival data and RNA-seq from at least one tumor (see Supplementary Data 15). For the 33 ovarian cancer patients, we first used a Cox proportional hazards model to control for patient age at diagnosis, tumor stage at diagnosis, *BRCA1* vs. *BRCA2* mutation, and disease recurrent status as confounding variables. We used the same confounding variables, in addition to ER status, in multivariate Cox proportional hazards analysis of the 34 breast cancer patients. In ovarian cancer patients, the only confounding variable associated with survival was disease recurrent status (a logical variable indicating whether the patient’s disease eventually recurred). Therefore, we simplified our ovarian Cox proportional hazards model to include recurrent status as the only confounding variable. In breast cancer patients, none of the confounding variables were significantly associated with survival, so these were discarded in favor of a univariate analysis. Cox proportional hazards analyses were performed and plotted using the Survival (v3.2.7) and Survminer (v0.4.8) R packages. *p*-values reported on the plots are those corresponding to the isoform expression term itself. *p*-values for the models were tested for significance by Wald test (*α* = 0.05, 1 df for breast, 2 df for ovarian).

### Reporting summary

Further information on research design is available in the Nature Portfolio Reporting Summary linked to this article.

## Supplementary information

Supplementary Information

Description of Additional Supplementary Information

Dataset 1

Dataset 2

Dataset 3

Dataset 4

Dataset 7

Dataset 6

Dataset 5

Dataset 8

Dataset 9

Dataset 10

Dataset 11

Dataset 12

Dataset 13

Dataset 14

Dataset 15

Dataset 16

Reporting Summary

## Data Availability

The raw capture-targeted, WES and RNA sequencing data generated as part of this study have been deposited in the NCBI SRA database under accession code PRJNA751555. The raw germline WES data are protected due to lack of patient consent to deposit in a public repository. The germline WES will be made available upon request from the corresponding author and will made available under a Data Transfer Agreement (DTA) and transferred via FTP when the DTA is complete. Most data transfers will be completed within a month’s time. Access to the raw imaging data also is available upon request from the corresponding author. TCGA data are available under controlled-use conditions; data use limitations and the instructions for applying for access are available through dbGaP. The raw TGCA data are accessible via the Genomic Data Commons from the National Center Institute (breast cancer patients from TCGA-BRCA, https://portal.gdc.cancer.gov/projects/TCGA-BRCA and ovarian cancer patients from TCGA-OV, https://portal.gdc.cancer.gov/projects/TCGA-OV) once access is made available through dbGAP application. All data needed to evaluate the conclusions in the paper are present in the paper and/or the Supplementary Information. Source Data are provided with this paper.

## Code availability

All code used for the study is available at https://github.com/nathanson-lab/BRCA-PrimaryRecurrent^115^. Additional code for calculating HRD and aneuploidy scores is available at https://github.com/maxwell-lab/HRDex.
