# Supplementary material for: Analysis of matched primary and recurrent BRCA1/2 mutation-associated tumors identifies recurrence-specific drivers
Source: Nat Commun. 2022 Nov 7;13:6728. doi: 10.1038/s41467-022-34523-y (PMC9640723; doi:10.1038/s41467-022-34523-y)
Supplement: Supplementary file 1 — Supplementary Information [file 41467_2022_34523_MOESM1_ESM.pdf]

Title: Analysis of matched primary and recurrent *BRCA1/2* mutation-associated tumors identifies recurrence-specific drivers

Supplementary Information Files

File name: Supplementary_information.pdf
Description: Supplementary Methods, Supplementary Tables, Supplementary Figures, Supplementary Figure Legends

File name: Supplementary_Data1.xlsx
Description: Clinical metadata and sequencing performed by tumor (Basser cohorts)

File name: Supplementary_Data2.xlsx
Description: Pathogenic mutations by sequencing type and by tumor group (primary-private, recurrence-private, and shared)

File name: Supplementary_Data3.xlsx
Description: Pathogenic *TP53* mutations by sequencing type and tumor group

File name: Supplementary_Data4.xlsx
Description: Tumor mutational burden, HRD, and aneuploidy scores by tumor

File name: Supplementary_Data5.xlsx
Description: Gene set enrichment analysis from whole exome sequencing (mutations and copy number variation)

File name: Supplementary_Data6.xlsx
Description: MutSigCV results from primary and recurrent tumor cohorts

File name: Supplementary_Data7.xlsx
Description: Targeted sequencing metrics, summary and for *BRCA1* and *BRCA2* genes

File name: Supplementary_Data8.xlsx
Description: Somatic Loss of Function Mutations in *BRCA1/2* by sequencing type and tumor group

File name: Supplementary_Data9.xlsx
Description: Results from all GISTIC analyses

File name: Supplementary_Data10.xlsx
Description: Subtractive analysis of GISTIC segments in primary vs. recurrent tumors

File name: Supplementary_Data11.xlsx
Description: *PARP1* copy number in primary/recurrent and TCGA cohorts

File name: Supplementary_Data12.xlsx
Description: TCGA tumors by group

File name: Supplementary_Data13.xlsx
Description: Gene set enrichment analysis from RNA sequencing

File name: Supplementary_Data14.xlsx
Description: Gene fusions (FusionInspector output) involving *MALAT1* and immunoglobulin genes

File name: Supplementary_Data15.xlsx
Description: *BRCA2* isoform expression by tumor and clinical metadata used for survival analyses; Cox Proportional Hazards models

File name: Supplementary_Data16.xlsx
Description: RT-qPCR results from validation of differential *BRCA2* isoform expression; primer sequences

Supplementary Methods

Validation of differential *BRCA2* transcript usage by quantitative reverse transcription PCR (RT-qPCR)

We validated expression of *BRCA2-001/Short* and *BRCA2-201/Long* by RT-qPCR in 20 primary and recurrent tumors with adequate RNA. For each sample, 1000 ng of RNA (without reverse transcription) was used as a genomic DNA contamination control (control sample) and another 1000 ng for reverse transcription and PCR amplification (experimental sample). Both control and experimental samples were treated with ezDNase (Invitrogen) to remove traces of genomic DNA (gDNA). Next, cDNA was generated from experimental RNA samples using Master Mix from the SuperScript IV VILO kit (Thermo Fisher); control samples were treated with the No RT Control mix from the same kit. Control samples did not undergo reversion transcription; otherwise, experimental and control samples were processed identically.

Quantitative PCR was performed on a QuantStudio Flex Real-Time PCR System (Applied Bio) using Power SYBR Green Master Mix (Thermo Fisher). *GAPDH* expression was used as the endogenous control. Three sets of primers were used to amplify and differentiate between *BRCA2-001/Short* and *BRCA2-201/Long*: one for the junction of Exon 26 and Exon 27 (Exon 26/27 primers), one for the 3' end of Exon 27 (Exon 27 primers), and one located in the intronic region between Exon 27 and Exon 28 of the long variant (Intron 27 primers). Our RNA sequencing data was used to guide the primer design, which supported expression in the three regions selected. As we used RNA exome (capture-based) expression data, we could determine that the segment of Intron 27 that we used for the primer design was included in the BRCA2-201/Long isoform, but not whether other portions or the entirety of the intron was expressed. All primer sequences are reported in **Supplementary Data 16** and depicted in **Supplementary Figure 10b.**

Five well were prepared for each sample on a 384-well plate: one for *GAPDH* expression in the experimental sample, three for each of the *BRCA2* primers in the experimental

sample, and one for *GAPDH* expression in the control sample. Each well was prepared with 10μL SYBR Green Master Mix, 6μL PCR-grade water, 1μL 10 μM forward primer, 1μL 10 μM reverse primer, and 2 μL cDNA. The PCR reaction program was: 50 °C for 2 minutes, 90 °C for 10 minutes for denaturing, 40 cycles of amplification (95 °C for 15 seconds then 59 °C for 60 seconds), and a final step with 95°C for 15 seconds and 50°C for 2 minutes. Two 384-well plates were run separately, for a total of two replicates per sample.

ΔCT values were calculated by subtracting the *GAPDH* CT value of the same sample from the CT value of *BRCA2* primer sets. Samples were excluded from further analysis if they failed to meet the following criteria in both replicates: 1) CT values ≤ 35 for all wells containing experimental RNA (*GAPDH* and *BRCA2* primers) and 2) CT values > 40 for contamination control RNA. ΔΔCT values were computed for the final cohort by normalizing ΔCT values to that of a single representative sample (**Supplementary Data 16**).

Construction of Tissue Microarrays (TMAs) containing primary and recurrent tumors

Twenty-three primary and recurrent *BRCA1/2* mutation-associated tumors (a subset of the primary/recurrent cohort used throughout the study) were assembled into three tissue microarrays (TMAs). Each tissue microarray contained 4-11 1mm cores per tumor to account for tumor heterogeneity, including intratumoral, stromal, and lymphocytic-rich areas. TMA blocks were constructed, cut, stained, and imaged at the Pathology Clinical Service Center at the University of Pennsylvania. Normal liver, spleen, and kidney tissue from other patients were included as controls for antibody testing.

Immunohistochemistry (IHC) analysis of PARP1

Immunohistochemistry for PARP1 was performed on each TMA using standard laboratory protocols and the PARP (46D11) Rabbit mAb (Cell Signaling Technology, catalog #9532). Stained slides were imaged for quantification at 20x resolution. PARP1 nuclear positivity was

quantified for each core using H-score (0-300 scale), as determined by AN. Hematoxylin and eosin (H&E) staining was used to assess tumor content in parallel; cores with inadequate tumor per H&E were excluded by AN. We calculated an average PARP1 H-score for each tumor for downstream analyses (range 3-10 evaluable cores per tumor).

<u>Co-detection by Indexing (CODEX) coverslip preparation and staining</u>

TMA slides were cut at 5µm and placed on previous coated poly-lysine-l coverslips for CODEX[37]. We used a custom 40-plex breast and ovarian cancer specific antibody panel (see **Supplementary Table 2** for antibody list including dilutions). CODEX staining was performed according to the manufacturer protocols, with one additional step to reduce background fluorescence[38]. Briefly, tissue coverslips were submerged in bleaching solution (4.5% (w/v) H_2O_2 and 20mM NaOH in PBS) after antigen retrieval. While in solution, slides were incubated between two LED lights (Aibecy A4 Ultra Bright 25,000 Lux LED Light) for 45min at room temperature (RT). Bleaching solution was then replaced, and tissue coverslips were incubated between lights for an additional 45min at RT. Coverslips were then washed four times in 1x PBS for 3-5 minutes. Lastly, the tissue was placed in staining solution following the manufacturer's protocol.

<u>CODEX Imaging</u>

Imaging was performed using a Leica DMi8 inverted microscope with 4 filter cubes (DAPI: Excitation: 359nm Emission: 457nm; RHOD: Excitation: 551nm, Emission: 573nm, Y5: Excitation: 651, Emission: 671nm; Y7: Excitation: 756nm, Emission: 779nm), equipped with 20x lens (numerical aperture: 0.8) and LAS X3.6.0.20104. Each core was imaged with a 20x immersion objective in a 2x2 tiled acquisition, ~300nm/pixel resolution and 6 z-planes per tile. CIM v1.29.0.364 was used to control Fluidics operation and set up cycle/marker information.

Processing of raw images from CODEX

Raw data were formatted and transferred using CIM version 1.29.0.364. We processed the data in order to perform cropping and stitching, flat field correction, drift-compensation, best focus definition and background subtraction. To remove out-of-focus light, image deconvolution was performed using Microvolution software (Microvolution LLC, California, USA). QPTIFFs were generated using processor v1.8.0.257 – dev. We generated 43-55 images per TMA (one per core), each consisting of 41 channels (40 antibodies plus 1 nuclear stain).

CODEX cell segmentation

Processed data (QPTIFF) was opened in Phenochart version 1.1.0. DAPI sensitivity was adapted in order to select individual core areas to train the segmentation algorithm. QPTIFF was then selected and loaded using InForm version 2.5.1. We adapted cell segmentation settings such as pixel intensity, nuclear component splitting, and expected cell size to detect cells with that varied in size and shape. To define single cell boundaries, we used three markers to identify tumor (TC) and immune cells (IC) and to distinguish between nuclei, cytoplasm and membrane: nuclear marker (DAPI), CD45 (IC), and PANCK (TC). All segmented areas were visually checked with ImageJ v1.53c to confirm appropriate segmentation.

CODEX marker quantification

We used ImageJ v1.53c with CODEX_MAV (version 1.5.0.8) plugin to visualize individual cores and merge cores by tumor. Samples were gated using CD45 to detect immune cells (IC), PANCK or Cytokeratin14 to detect tumor cells (TC), and Vimentin to detected stromal cells. Individual populations for each patient were then gated on RAD51, PD-1 and CTLA-4 positivity. Additionally, pixel intensity of each marker was determined for each population (normalized counts) using ImageJ MAV version 1.5.0.8. All gated cells of each population (such as PANCK+/RAD51+ cells) were visually assessed using ImageJ MAV. The percentages of

RAD51+, CTLA-4+, and PD-1+ cells per population (TC or IC) were calculated using the total cell number of each population. In regards to select RAD51+ cells, we only included cells with a nuclear RAD51 expression <1-2μm. We visualized groupwise percentages of cells with box plots generated with GraphPad Prism 6.0. Groupwise differences were assessed using two-sided Wilcoxon rank sum tests (α=0.05).

Supplementary Tables

Supplementary Table 1: Genes included in targeted capture for high-depth sequencing, by coverage type.

Exons+UTR Coverage				Whole Gene Coverage	
ABL1	DICER1	MAP2K2	RAD51	ABCB1	TGFBR2
AKT1	DIRAS3	MAP2K4	RAD51B	APC	TOP2B
AKT2	DLEC1	MAP3K4	RAD51C	ATXN3	TP53
AKT3	DPH1	MAPK1	RAD51D	BRCA1	TSC1
ALK	EIF5A2	MAPK3	RAD52	BRCA2	TSC2
AQR	EME1	MECOM	RAD54B	CCNE1	XRCC1
ARAF	EME2	MET	RAD54L	CDH1	
ARID1A	EPHA3	MLH1	RASSF1	CHD4	
ARL11	ERBB2	MPL	RFC1	CUL4B	
ATM	ERBB3	MRE11A	RNF111	EGFR	
ATR	ERBB4	MSH3	RP11-554I8.2	EP300	
AURKA	ERCC2	MSH4	RP3-510D11.2	FGFR2	
BAP1	ERCC3	MSH6	RPA1	FGFR3	
BARD1	ERCC4	MTOR	RPA2	FLT4	
BLM	ERCC6	MUS81	RPA3	FOXA1	
BRAF	ESR1	NBN	RPA4	GATA3	
BRIP1	FANCA	NF2	RPS6KA2	JAK1	
C11ORF80	FANCE	NOTCH2	RSF1	MAD2L2	
CBX2	FANCM	NOTCH3	SETMAR	MAP3K1	
CCND1	FBXW7	NRAS	SHFM1	MDM2	
CCND2	FGF1	OPCML	SLX4	MSH2	
CCND3	FGFR1	PALB2	SMAD2	MYC	
CDK12	FLT3	PAX8	SMARCA4	NCOR1	
CDK4	FOXL2	PEG3	SMARCB1	NF1	
CDK6	FRS2	PIK3R1	SMCHD1	NOTCH1	
CDKN1A	GNA11	PLAGL1	SPARC	PARP1	
CDKN1B	GNAQ	PMS2	SRC	PARP2	
CDKN1C	GNAS	POLD1	SSBP1	PDGFRA	
CDKN2A	GTF2H3	POLD2	STK11	PIK3CA	
CDKN2B	HRAS	POLD3	TERT	POLE	
CEBPA	IGF1R	POLD4	TOP2A	POLR2A	
CHEK1	JAK2	POLK	TOP3A	PRDM7	
CHEK2	JAK3	PPIE	TOP3B	PTEN	
CREBBP	JARID2	PPM1D	TP53BP1	RAD23B	
CRKL	KDR	PPP2R1A	TRIP13	RAF1	
CSF1R	KIT	PRDM9	WWOX	RB1	
CTNNB1	KLHDC3	PRKCI	XRCC2	RBBP8	
CUL4A	KMT2C	PRKDC	XRCC3	RIC8A	
DAB2	KRAS	PRPF19	XRCC6	RIF1	
DDB1	LIG3	RAB25		SMAD4	
DDR2	MAP2K1	RAD50		TBX3	

Supplementary Table 2: Antibodies used for CODEX.

Antibody	Clone	Dilution	Manufacturer
Anti-human pATM	EP1890Y	1:50	Abcam
Anti-human RAD51	EPR4030(3)	1:50	Abcam
Anti-human PD-1	D4W2J	1:100	Cell Signaling
Anti-human BRCA1	MS110	1:100	Abcam
Anti-human CTLA-4	EPR1476	1:100	Abcam
Anti-human CD45	2B11+PD7/26	1:100	Novusbio
Anti-human PanCK	AE-1/AE-3	1:200	Akoya
Anti-human Keratin 14	Poly19053	1:200	Akoya
Anti-human H3-Ser28	HTA28	1:200	Akoya

Supplementary Figures and Figure Legends

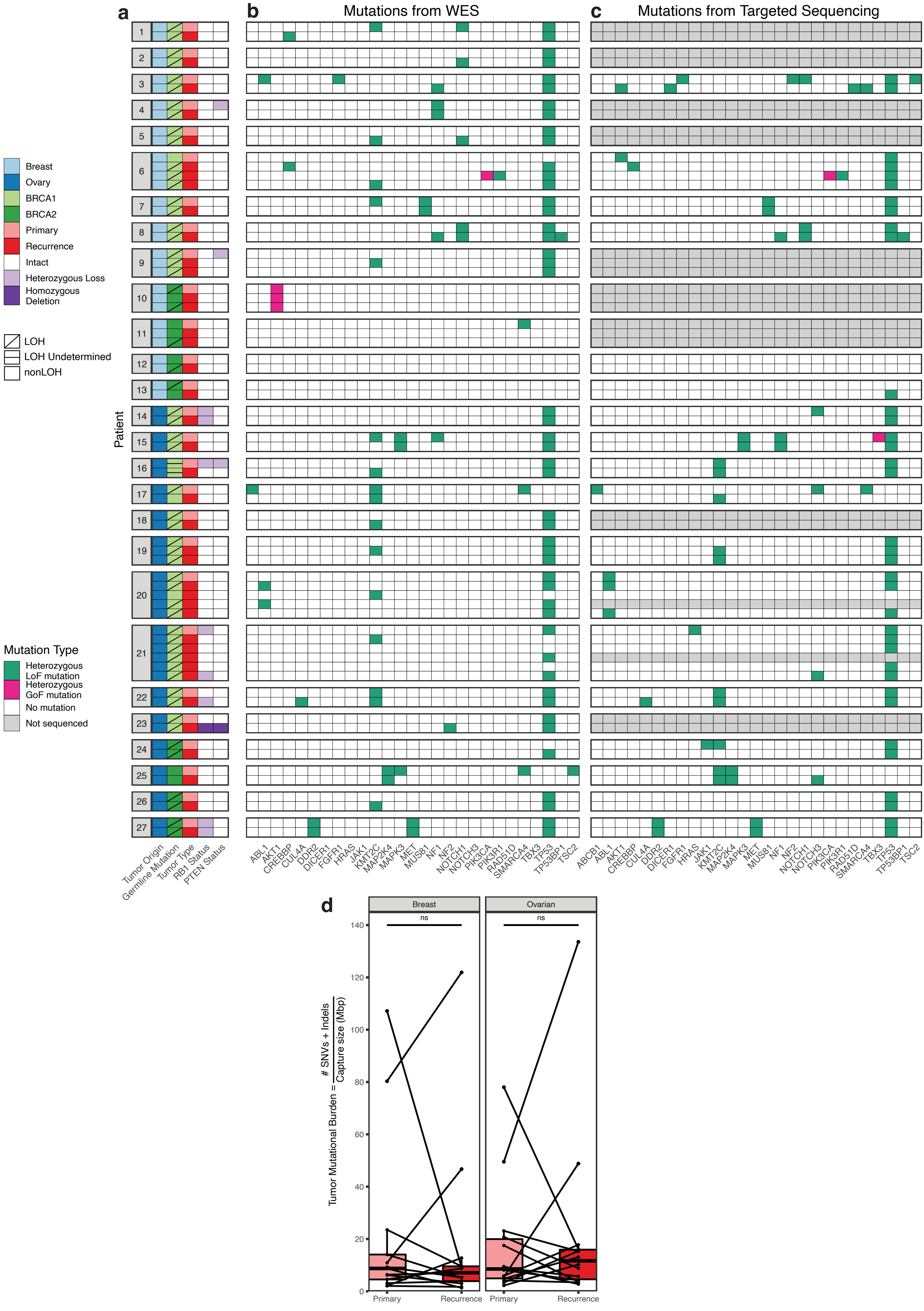

a
b
Mutations from WES
c
Mutations from Targeted Sequencing
Breast
Ovary
BRCA1
BRCA2
Primary
Recurrence
Intact
Heterozygous Loss
Homozygous Deletion
LOH
LOH Undetermined
nonLOH
Mutation Type
Heterozygous LoF mutation
Heterozygous GoF mutation
No mutation
Not sequenced
Patient
Tumor Origin
Germline Mutation
Tumor Type
RB1 Status
PTEN Status
ABL1 AKT1 CREBBP CUL4A DDR2 DICER1 FGFR1 HRAS JAK1 KMT2C MAP2K4 MAPK3 MET MUS81 NF1 NF2 NOTCH1 NOTCH3 PIK3CA PIK3R1 RAD51D SMARCA4 TBX3 TP53 TP53BP1 TSC2
ABCB1 ABL1 AKT1 CREBBP CUL4A DDR2 DICER1 FGFR1 HRAS JAK1 KMT2C MAP2K4 MAPK3 MET MUS81 NF1 NF2 NOTCH1 NOTCH3 PIK3CA PIK3R1 RAD51D SMARCA4 TBX3 TP53 TP53BP1 TSC2
d
Breast
Ovarian
ns
Tumor Mutational Burden = # SNVs + Indels / Capture size (Mbp)
Primary
Recurrence

Supplementary Figure 1. Stratified analysis of mutations from whole exome and targeted sequencing. A. 67 paired primary and recurrent tumors from 27 patients sequenced by whole exome sequencing (WES, n=67) and high-depth targeted sequencing (n=44). Tumors are displayed in chronological order by patient, with the primary tumor at the top and latest recurrence at the bottom. LOH = *BRCA1/2* allele-specific loss of heterozygosity. B. Somatic mutations from WES, by mutation type and zygosity. C. Somatic mutations from targeted sequencing, by mutation type and zygosity. Display in B and C is limited to genes with ≥ 1 mutation with alternative allele fraction ≥0.05 from targeted sequencing. D. Comparison of tumor mutational burden for primary/recurrent tumor pairs (n = 54 biologically independent tumor samples, comprising 2 samples/patient from 27 patients). For patients with multiple recurrences, one recurrence was chosen at random for comparison. Boxplot elements are as follows: median, center line; box limits, first and third quartiles (spanning the IQR, interquartile range); whiskers, 1.5x IQR in each direction; outliers plotted individually. Pairwise differences were determined by two-sided Wilcoxon signed rank test (α=0.05). NS = not significant.

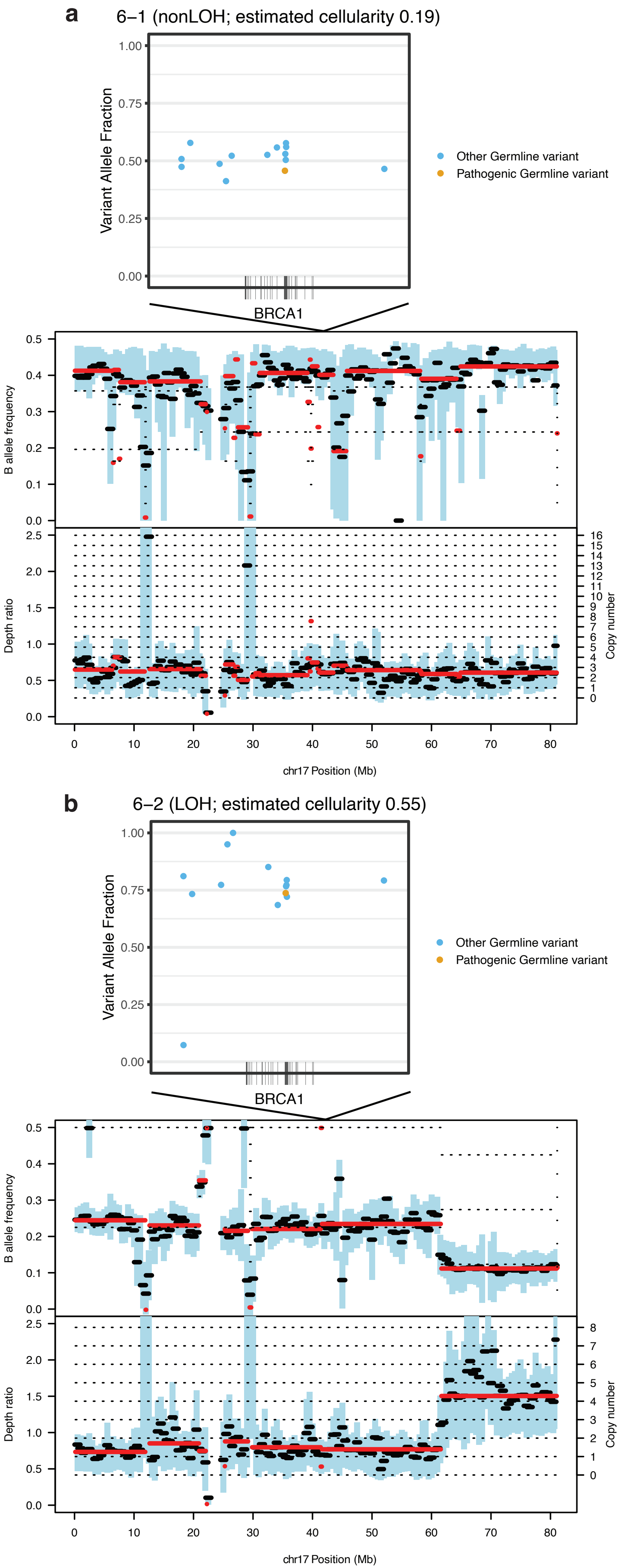

a
6–1 (nonLOH; estimated cellularity 0.19)
Variant Allele Fraction
1.00
0.75
0.50
0.25
0.00
Other Germline variant
Pathogenic Germline variant
BRCA1
B allele frequency
Depth ratio
Copy number
chr17 Position (Mb)
b
6–2 (LOH; estimated cellularity 0.55)
Variant Allele Fraction
Other Germline variant
Pathogenic Germline variant
BRCA1
B allele frequency
Depth ratio
Copy number
chr17 Position (Mb)

Supplementary Figure 2. *BRCA1* germline variants, B allele frequency, and copy number before and after a representative nonLOH to LOH transition. A. Location and variant allele fraction of *BRCA1* germline variants in Patient 6's primary breast tumor without allele-specific loss of heterozygosity LOH (top), pictured with B allele frequency and copy number across chromosome 17 (bottom). B. Location and variant allele fraction of *BRCA1* germline variant in Patient 6's first breast tumor recurrence with LOH (top), pictured with B allele frequency and copy number across chromosome 17 (bottom). For A and B, germline variants were called by VarDictJava and VarScan2. B allele frequency and copy number plots were generated in Sequenza.

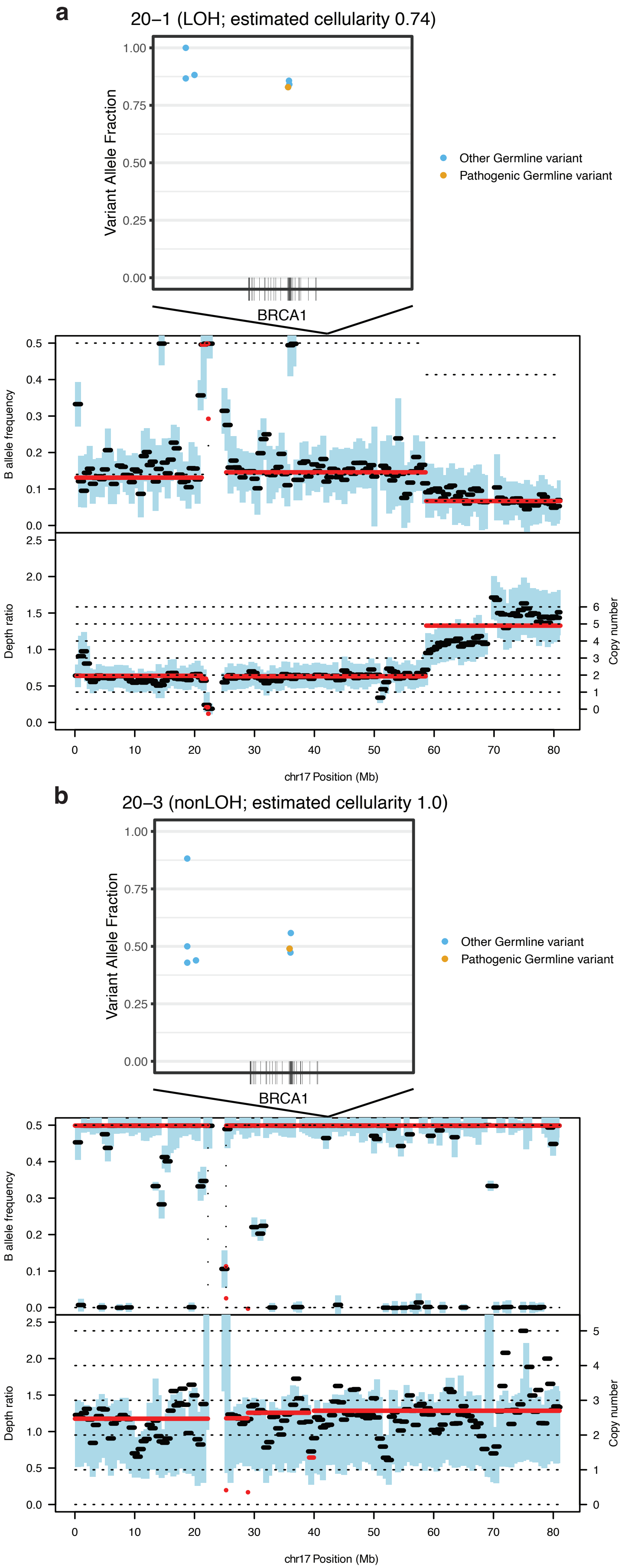

a
20−1 (LOH; estimated cellularity 0.74)
Variant Allele Fraction
1.00
0.75
0.50
0.25
0.00
BRCA1
Other Germline variant
Pathogenic Germline variant
B allele frequency
Depth ratio
Copy number
chr17 Position (Mb)
b
20−3 (nonLOH; estimated cellularity 1.0)
Variant Allele Fraction
BRCA1
Other Germline variant
Pathogenic Germline variant
B allele frequency
Depth ratio
Copy number
chr17 Position (Mb)

Supplementary Figure 3. *BRCA1* germline variants, B allele frequency, and copy number before and after a representative LOH reversal. A. Location and variant allele fraction of *BRCA1* germline variants in Patient 20's primary ovarian tumor with allele-specific loss of heterozygosity (LOH, top), pictured with B allele frequency and copy number across chromosome 17 (bottom). B. Location and variant allele fraction of *BRCA1* germline variant in Patient 20's second ovarian tumor recurrence without LOH (top), pictured with B allele frequency and copy number across chromosome 17 (bottom). For A and B, germline variants were called by VarDictJava and VarScan2. B allele frequency and copy number plots were generated in Sequenza.

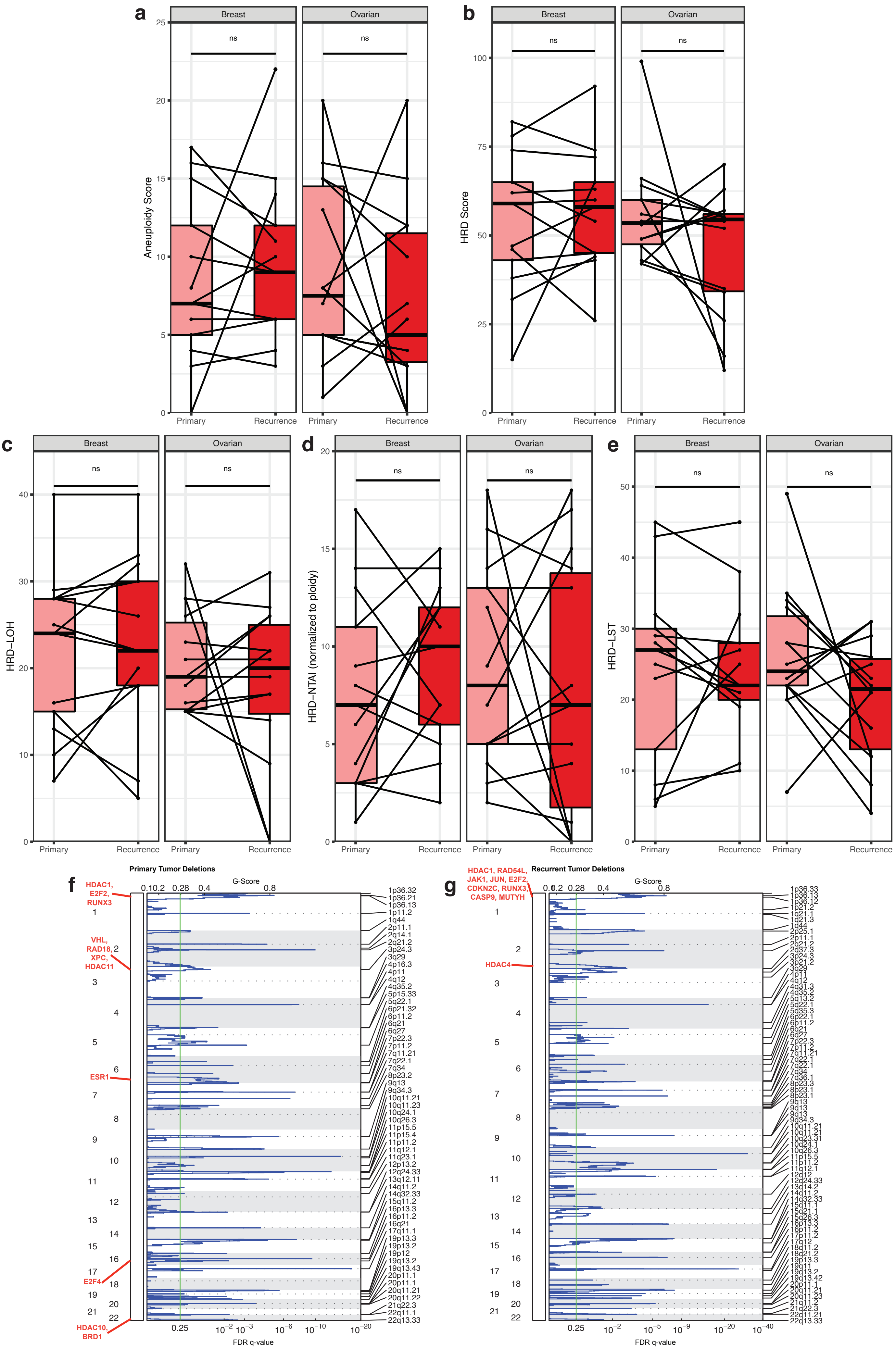

a
Breast
Ovarian
ns
Aneuploidy Score
Primary
Recurrence
b
HRD Score
c
HRD−LOH
d
HRD−NTAI (normalized to ploidy)
e
HRD−LST
f
Primary Tumor Deletions
G-Score
FDR q-value
HDAC1, E2F2, RUNX3
VHL, RAD18, XPC, HDAC11
ESR1
E2F4
HDAC10, BRD1
1p36.32
1p36.21
1p36.13
1p11.2
1q44
2p11.1
2q14.1
2q21.2
3p24.3
3q29
4p16.3
4p11
4q12
4q35.2
5p15.33
5q22.1
6p21.32
6p11.2
6q21
6q27
7p22.3
7p11.2
7q11.21
7q22.1
7q34
8p23.2
9q13
9q34.3
10q11.21
10q11.23
10q24.1
10q26.3
11p15.5
11p15.4
11p11.2
11q12.1
11q23.1
12p13.2
12q24.33
13q12.11
14q11.2
14q32.33
15q11.2
16p13.3
16p11.2
16q21
17q11.1
19p13.3
19p13.2
19p12
19q13.2
19q13.43
20p11.1
20p11.1
20q11.21
20q11.22
21q22.3
22q11.1
22q13.33
g
Recurrent Tumor Deletions
HDAC1, RAD54L, JAK1, JUN, E2F2, CDKN2C, RUNX3, CASP9, MUTYH
HDAC4

Supplementary Figure 4. Genome-wide measures of copy number variation in primary/recurrent cohort. A. Comparison of aneuploidy score for primary/recurrent tumor pairs (n = 54 biologically independent tumor samples, comprising 2 samples/patient from 27 patients). B. Comparison of Homologous Recombination Deficiency (HRD) score for primary/recurrent tumor pairs (n = 54 biologically independent tumor samples, comprising 2 samples/patient from 27 patients). C-E. Comparison of individual HRD metrics for primary/recurrent tumor pairs (n = 54 biologically independent tumor samples, comprising 2 samples/patient from 27 patients): loss of heterozygosity (HRD-LOH, C), non-telomeric allelic imbalance (HRD-NTAI, D), and large scale state transitions (HRD-LST, E). For patients with multiple recurrences, one recurrence was chosen at random for statistical comparisons in plots A-E. Boxplot elements are as follows: median, center line; box limits, first and third quartiles (spanning the IQR, interquartile range); whiskers, 1.5x IQR in each direction; outliers plotted individually. Pairwise differences were determined by paired two-sided Wilcoxon signed rank test (α=0.05). F. GISTIC qplot for 90% confidence interval deletions in primary tumors. G. GISTIC qplot for 90% confidence interval deletions in recurrences. For F and G, all highlighted genes have residual q<0.05. NS = not significant.

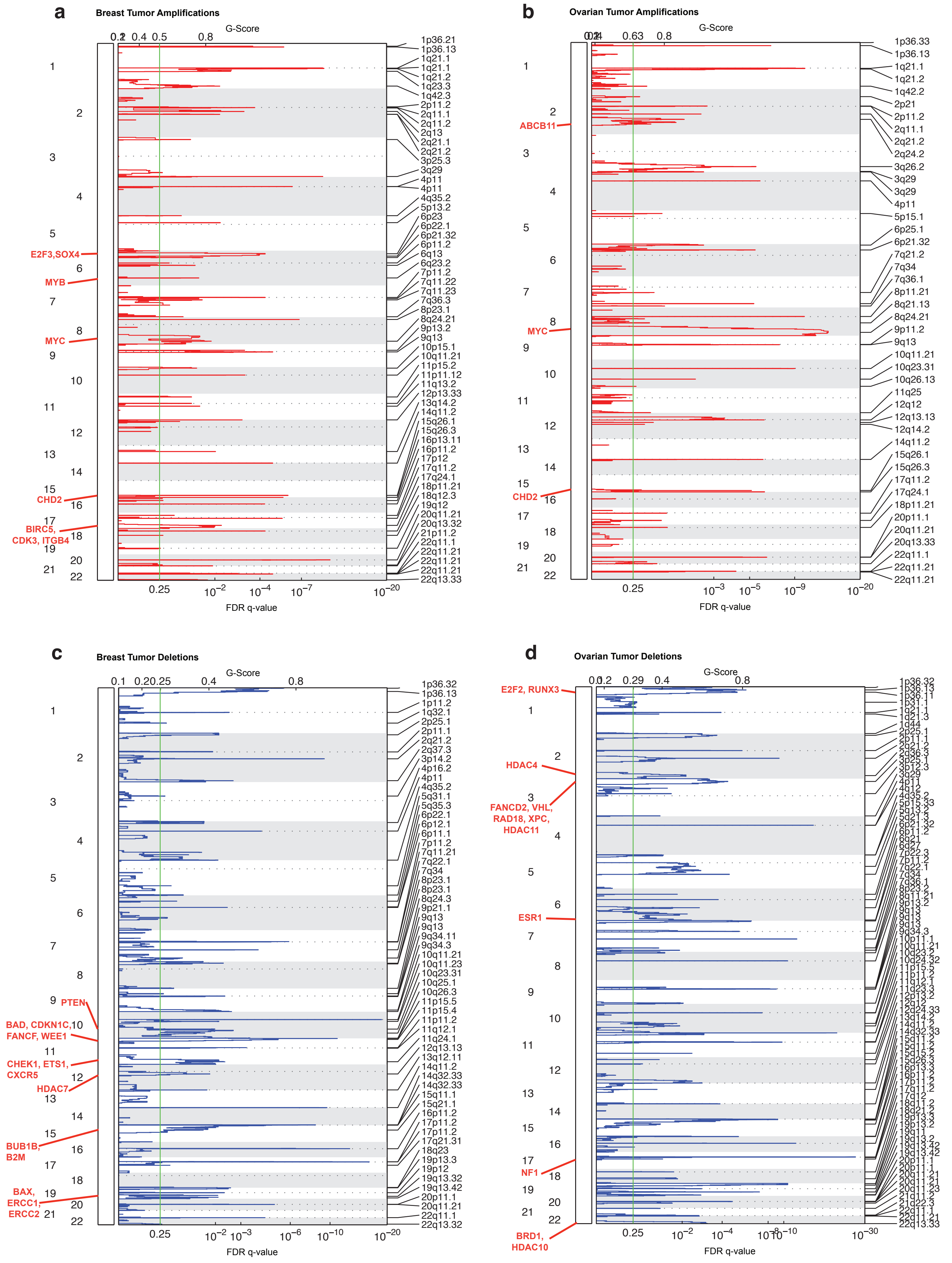
a
Breast Tumor Amplifications
G-Score
0.1 0.2 0.4 0.5 0.8
E2F3,SOX4
MYB
MYC
CHD2
BIRC5, CDK3, ITGB4
1p36.21
1p36.13
1q21.1
1q21.1
1q21.2
1q23.3
1q42.3
2p11.2
2q11.1
2q11.2
2q13
2q21.1
2q21.2
3p25.3
3q29
4p11
4p11
4q35.2
5p13.2
6p23
6p22.1
6p21.32
6p11.2
6q13
6q23.2
7p11.2
7q11.22
7q11.23
7q36.3
8p23.1
8q24.21
9p13.2
9q13
10p15.1
10q11.21
11p15.2
11p11.12
11q13.2
12p13.33
13q14.2
14q11.2
15q26.1
15q26.3
16p13.11
16p11.2
17p12
17q11.2
17q24.1
18p11.21
18q12.3
19q12
20q11.21
20q13.32
21p11.2
22q11.1
22q11.21
22q11.21
22q11.21
22q13.33
0.25 10^{-2} 10^{-4} 10^{-7} 10^{-20}
FDR q-value
b
Ovarian Tumor Amplifications
G-Score
0.1 0.24 0.63 0.8
ABCB11
MYC
CHD2
1p36.33
1p36.13
1q21.1
1q21.2
1q42.2
2p21
2p11.2
2q11.1
2q21.2
2q24.2
3q26.2
3q29
3q29
4p11
5p15.1
6p25.1
6p21.32
7q21.2
7q34
7q36.1
8p11.21
8q21.13
8q24.21
9p11.2
9q13
10q11.21
10q23.31
10q26.13
11q25
12q12
12q13.13
12q14.2
14q11.2
15q26.1
15q26.3
17q11.2
17q24.1
18p11.21
20p11.1
20q11.21
20q13.33
22q11.1
22q11.21
22q11.21
0.25 10^{-3} 10^{-5} 10^{-9} 10^{-20}
FDR q-value
c
Breast Tumor Deletions
G-Score
0.1 0.2 0.25 0.4 0.8
PTEN
BAD, CDKN1C, FANCF, WEE1
CHEK1, ETS1, CXCR5
HDAC7
BUB1B, B2M
BAX, ERCC1, ERCC2
1p36.32
1p36.13
1p11.2
1q32.1
2p25.1
2p11.1
2q21.2
2q37.3
3p14.2
4p16.2
4p11
4q35.2
5q31.1
5q35.3
6p22.1
6p12.1
6p11.1
7p11.2
7q11.21
7q22.1
7q34
8p23.1
8p23.1
8q24.3
9p21.1
9q13
9q13
9q34.11
9q34.3
10q11.21
10q11.23
10q23.31
10q25.1
10q26.3
11p15.5
11p15.4
11p11.2
11q12.1
11q24.1
12q13.13
13q12.11
14q11.2
14q32.33
14q32.33
15q11.1
15q21.1
16p11.2
17p11.2
17p11.2
17q21.31
18q23
19p13.3
19p12
19q13.32
19q13.42
20p11.1
20q11.21
22q11.1
22q13.32
0.25 10^{-2} 10^{-3} 10^{-6} 10^{-10} 10^{-20}
FDR q-value
d
Ovarian Tumor Deletions
G-Score
0.1 0.2 0.29 0.4 0.8
E2F2, RUNX3
HDAC4
FANCD2, VHL, RAD18, XPC, HDAC11
ESR1
NF1
BRD1, HDAC10
0.25 10^{-2} 10^{-4} 10^{-8} 10^{-10} 10^{-30}
FDR q-value

Supplementary Figure 5. GISTIC results by tumor type in primary/recurrent cohort. A. GISTIC qplot for 90% confidence interval amplifications in (primary and recurrent) breast tumors. B. GISTIC qplot for 90% confidence interval amplifications in (primary and recurrent) ovarian tumors. C. GISTIC qplot for 90% confidence interval deletions in (primary and recurrent) breast tumors. D. GISTIC qplot for 90% confidence interval deletions in (primary and recurrent) ovarian tumors. For A-D, all highlighted genes have residual q<0.05.

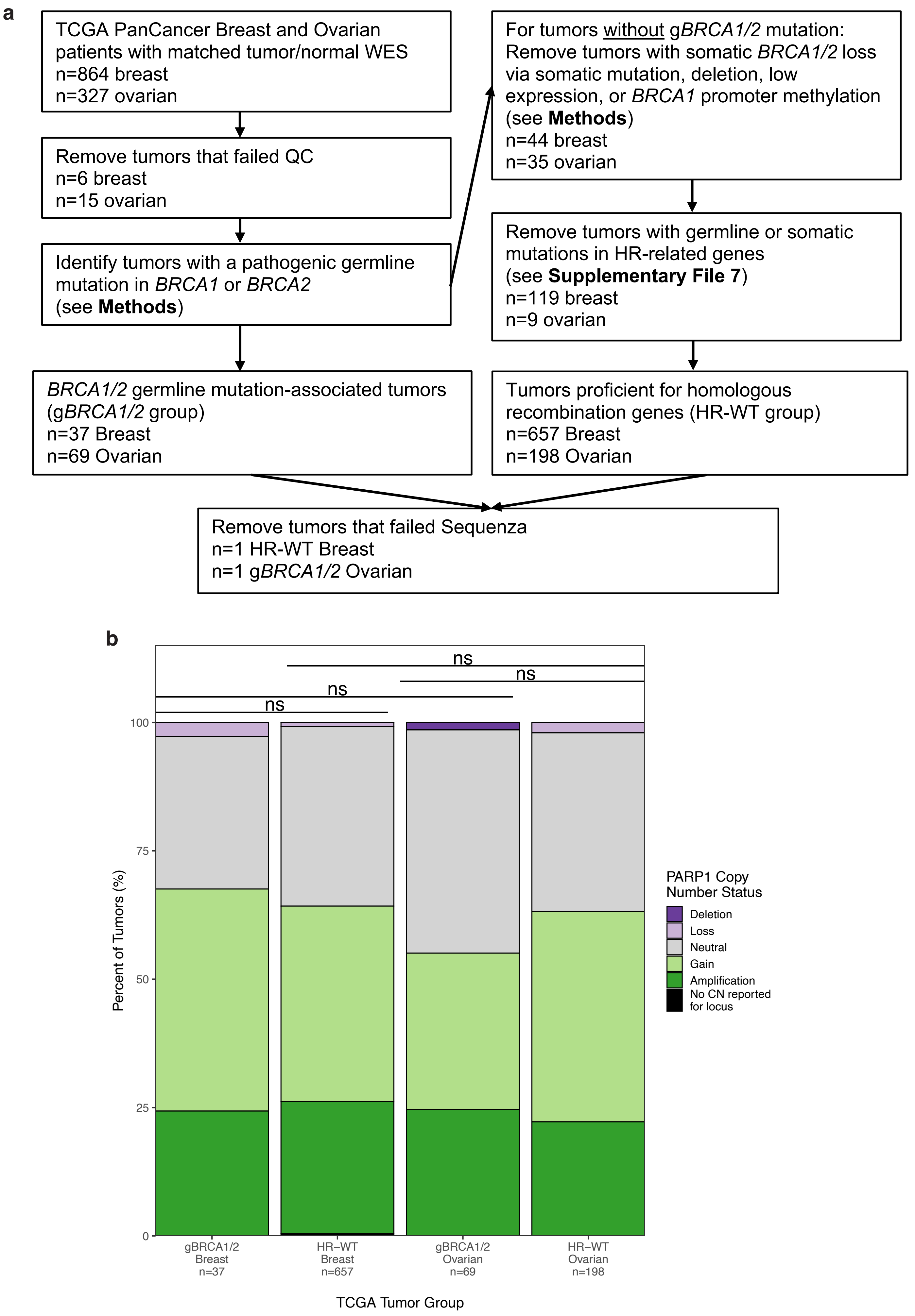
a
TCGA PanCancer Breast and Ovarian patients with matched tumor/normal WES n=864 breast n=327 ovarian
Remove tumors that failed QC n=6 breast n=15 ovarian
Identify tumors with a pathogenic germline mutation in BRCA1 or BRCA2 (see Methods)
BRCA1/2 germline mutation-associated tumors (gBRCA1/2 group) n=37 Breast n=69 Ovarian
For tumors without gBRCA1/2 mutation: Remove tumors with somatic BRCA1/2 loss via somatic mutation, deletion, low expression, or BRCA1 promoter methylation (see Methods) n=44 breast n=35 ovarian
Remove tumors with germline or somatic mutations in HR-related genes (see Supplementary File 7) n=119 breast n=9 ovarian
Tumors proficient for homologous recombination genes (HR-WT group) n=657 Breast n=198 Ovarian
Remove tumors that failed Sequenza n=1 HR-WT Breast n=1 gBRCA1/2 Ovarian
b
ns
ns
ns
ns
100
75
50
25
0
Percent of Tumors (%)
gBRCA1/2 Breast n=37
HR−WT Breast n=657
gBRCA1/2 Ovarian n=69
HR−WT Ovarian n=198
TCGA Tumor Group
PARP1 Copy Number Status
Deletion
Loss
Neutral
Gain
Amplification
No CN reported for locus

Supplementary Figure 6. Analysis of *PARP1* copy number variation in TCGA cohorts. A. Strategy for grouping The Cancer Genome Atlas (TCGA) breast and ovarian tumors into those with germline *BRCA1/2* mutations (g*BRCA1/2*) and “Homologous Recombination-Wild Type” (HR-WT) groups for analysis. Tumors were excluded on the basis of somatic *BRCA1/2* loss or mutations in genes required for homologous recombination. B. *PARP1* copy number by tumor in selected TCGA breast and ovarian cohorts. Groupwise differences in average copy number were determined by two-sided t-test (α=0.05). Copy number (CN, from Sequenza) was binned as follows: Deletion, CN=0; Loss, CN=1; Neutral, CN=2-3; Gain, CN=4-5; Amplification, CN≥6. NS = not significant.

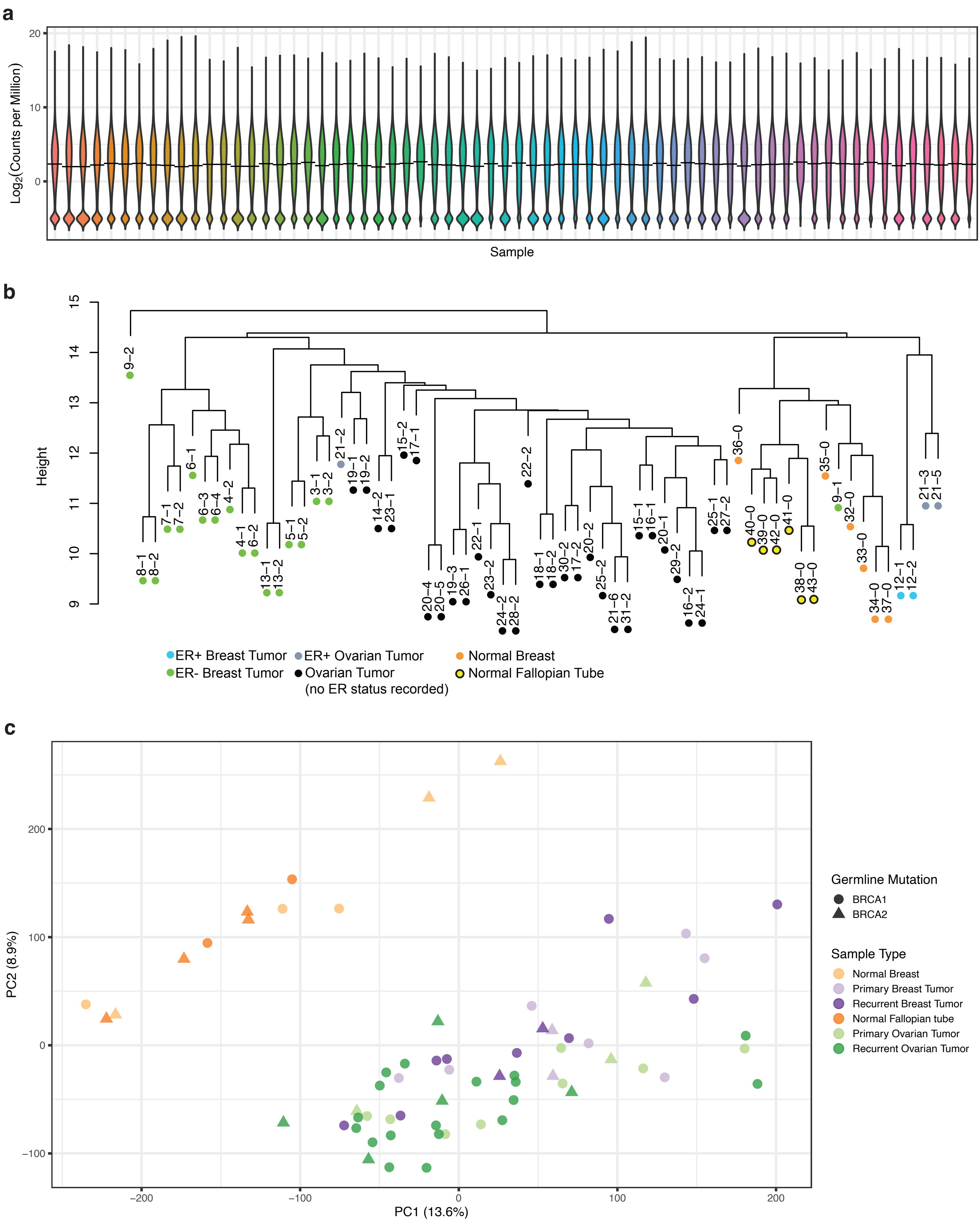
a
Log2(Counts per Million)
Sample
b
Height
ER+ Breast Tumor
ER+ Ovarian Tumor
Normal Breast
ER- Breast Tumor
Ovarian Tumor
(no ER status recorded)
Normal Fallopian Tube
c
PC2 (8.9%)
PC1 (13.6%)
Germline Mutation
BRCA1
BRCA2
Sample Type
Normal Breast
Primary Breast Tumor
Recurrent Breast Tumor
Normal Fallopian tube
Primary Ovarian Tumor
Recurrent Ovarian Tumor

Supplementary Figure 7. Sample relatedness identified by RNA sequencing. A. Distribution of log_2(counts per million) across cohort of samples used for RNA sequencing. B. Dendrogram of sample relatedness across RNA sequencing cohort, by tumor type and estrogen receptor (ER) status. C. Principal components (PC) analysis of RNA sequencing cohort.

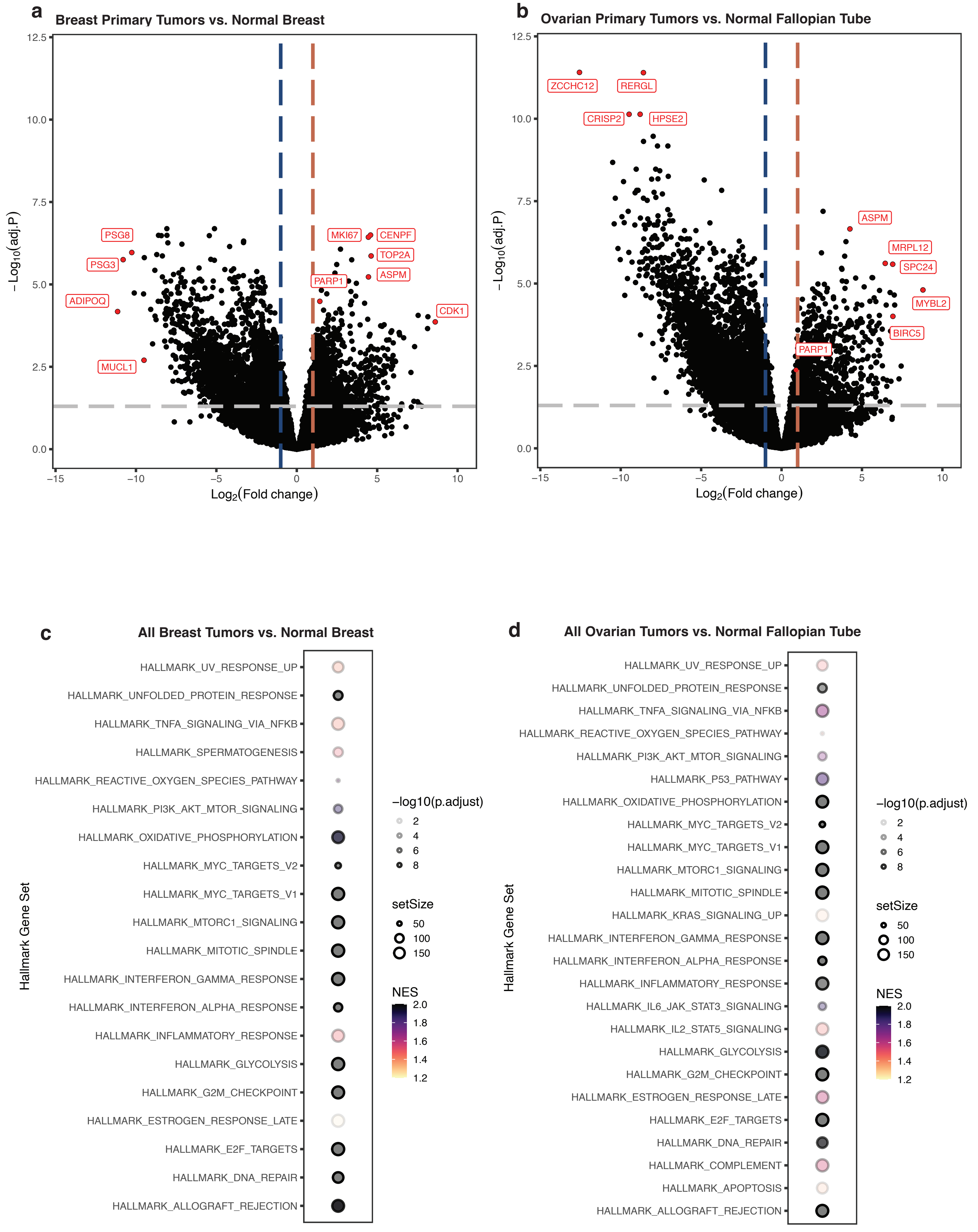
a
Breast Primary Tumors vs. Normal Breast
−Log10(adj.P)
Log2(Fold change)
PSG8
PSG3
ADIPOQ
MUCL1
MKI67
CENPF
TOP2A
ASPM
PARP1
CDK1
b
Ovarian Primary Tumors vs. Normal Fallopian Tube
ZCCHC12
RERGL
CRISP2
HPSE2
ASPM
MRPL12
SPC24
MYBL2
BIRC5
PARP1
c
All Breast Tumors vs. Normal Breast
Hallmark Gene Set
HALLMARK_UV_RESPONSE_UP
HALLMARK_UNFOLDED_PROTEIN_RESPONSE
HALLMARK_TNFA_SIGNALING_VIA_NFKB
HALLMARK_SPERMATOGENESIS
HALLMARK_REACTIVE_OXYGEN_SPECIES_PATHWAY
HALLMARK_PI3K_AKT_MTOR_SIGNALING
HALLMARK_OXIDATIVE_PHOSPHORYLATION
HALLMARK_MYC_TARGETS_V2
HALLMARK_MYC_TARGETS_V1
HALLMARK_MTORC1_SIGNALING
HALLMARK_MITOTIC_SPINDLE
HALLMARK_INTERFERON_GAMMA_RESPONSE
HALLMARK_INTERFERON_ALPHA_RESPONSE
HALLMARK_INFLAMMATORY_RESPONSE
HALLMARK_GLYCOLYSIS
HALLMARK_G2M_CHECKPOINT
HALLMARK_ESTROGEN_RESPONSE_LATE
HALLMARK_E2F_TARGETS
HALLMARK_DNA_REPAIR
HALLMARK_ALLOGRAFT_REJECTION
−log10(p.adjust)
setSize
NES
d
All Ovarian Tumors vs. Normal Fallopian Tube
Hallmark Gene Set
HALLMARK_UV_RESPONSE_UP
HALLMARK_UNFOLDED_PROTEIN_RESPONSE
HALLMARK_TNFA_SIGNALING_VIA_NFKB
HALLMARK_REACTIVE_OXYGEN_SPECIES_PATHWAY
HALLMARK_PI3K_AKT_MTOR_SIGNALING
HALLMARK_P53_PATHWAY
HALLMARK_OXIDATIVE_PHOSPHORYLATION
HALLMARK_MYC_TARGETS_V2
HALLMARK_MYC_TARGETS_V1
HALLMARK_MTORC1_SIGNALING
HALLMARK_MITOTIC_SPINDLE
HALLMARK_KRAS_SIGNALING_UP
HALLMARK_INTERFERON_GAMMA_RESPONSE
HALLMARK_INTERFERON_ALPHA_RESPONSE
HALLMARK_INFLAMMATORY_RESPONSE
HALLMARK_IL6_JAK_STAT3_SIGNALING
HALLMARK_IL2_STAT5_SIGNALING
HALLMARK_GLYCOLYSIS
HALLMARK_G2M_CHECKPOINT
HALLMARK_ESTROGEN_RESPONSE_LATE
HALLMARK_E2F_TARGETS
HALLMARK_DNA_REPAIR
HALLMARK_COMPLEMENT
HALLMARK_APOPTOSIS
HALLMARK_ALLOGRAFT_REJECTION
−log10(p.adjust)
setSize
NES

Supplementary Figure 8. Differential gene expression in tumors vs. normal tissue. A. Differential gene expression in primary breast tumors vs. normal breast tissue from *BRCA1/2* mutation carriers. B. Differential gene expression in primary ovarian tumors vs. normal fallopian tube tissue from *BRCA1/2* mutation carriers. For A and B, a positive Log_2(fold change) indicates genes with increased expression in primary tumors. Adjusted p values were computed based on linear modeling of mean-variance trends (limma). C. Hallmark Gene Sets enriched in genes with increased expression in primary and recurrent breast tumors compared to normal breast tissue from *BRCA1/2* mutation carriers. D. Hallmark Gene Sets enriched in genes with increased expression in primary and recurrent ovarian tumors compared to normal fallopian tube from *BRCA1/2* mutation carriers. For C and D, all gene sets had adj. $p<0.05$. NES = normalized enrichment score.

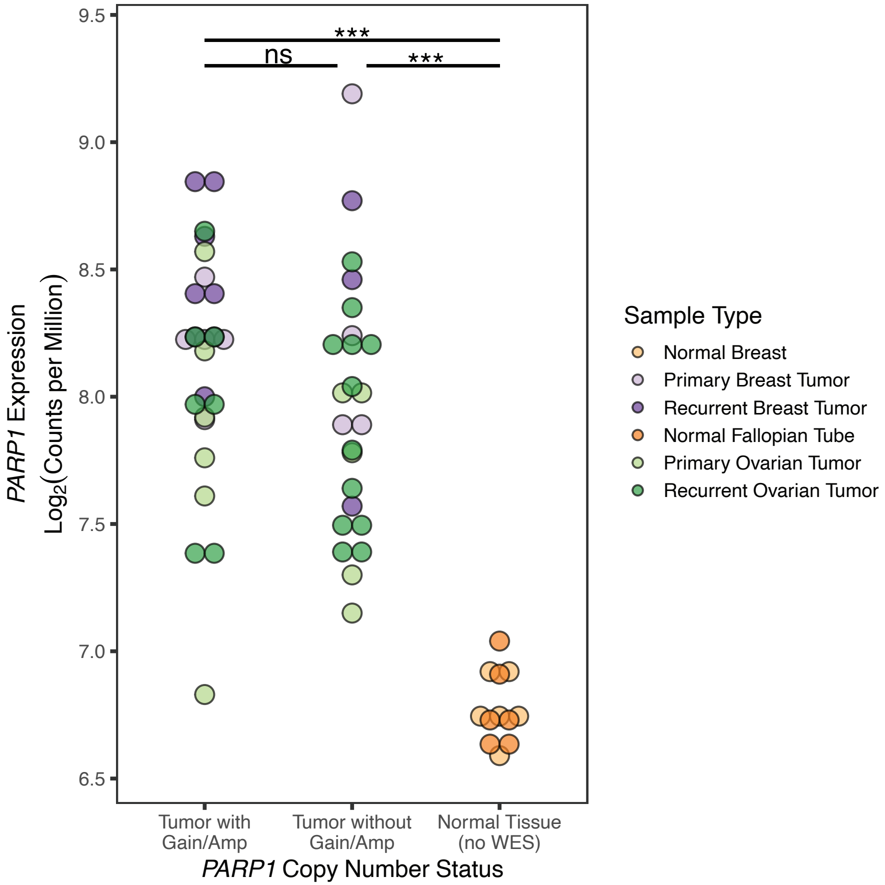

***
ns
***
9.5
9.0
8.5
8.0
7.5
7.0
6.5
PARP1 Expression
Log2(Counts per Million)
Tumor with Gain/Amp
Tumor without Gain/Amp
Normal Tissue (no WES)
PARP1 Copy Number Status
Sample Type
Normal Breast
Primary Breast Tumor
Recurrent Breast Tumor
Normal Fallopian Tube
Primary Ovarian Tumor
Recurrent Ovarian Tumor

Supplementary Figure 9. *PARP1* expression and copy number status in primary and recurrent tumors. *PARP1* copy number status and expression across all tumors with whole exome and RNA sequencing, and all normal samples with RNA-seq. Groupwise differences in average copy number were determined by Kruskal-Wallis test, followed by Dunn's test with Bonferroni correction (α=0.05, ***p<0.0001). NS = not significant.

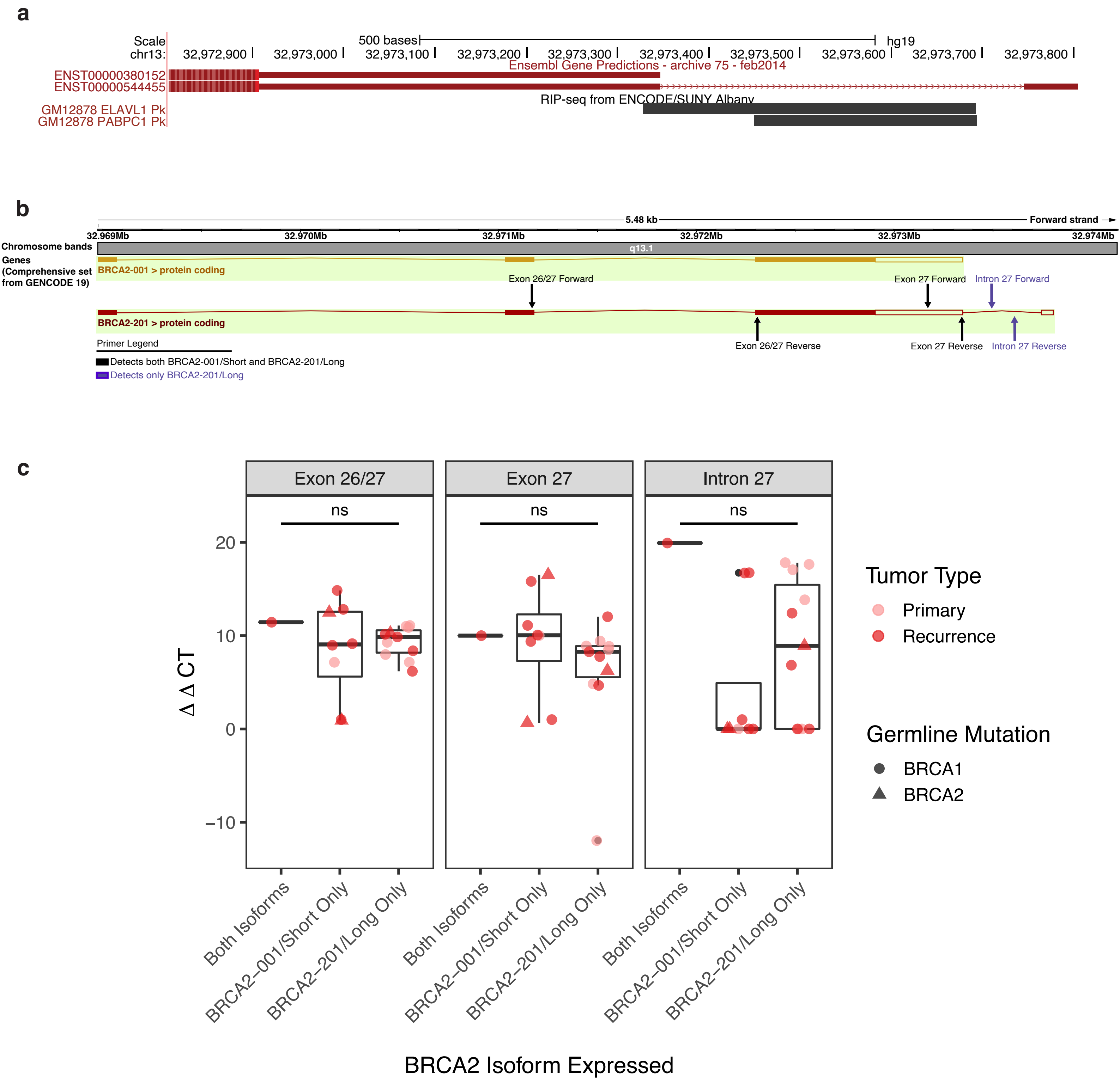

a
Scale
chr13:
500 bases
hg19
32,972,900
32,973,000
32,973,100
32,973,200
32,973,300
32,973,400
32,973,500
32,973,600
32,973,700
32,973,800
Ensembl Gene Predictions - archive 75 - feb2014
ENST00000380152
ENST00000544455
RIP-seq from ENCODE/SUNY Albany
GM12878 ELAVL1 Pk
GM12878 PABPC1 Pk
b
5.48 kb
Forward strand
32.969Mb
32.970Mb
32.971Mb
32.972Mb
32.973Mb
32.974Mb
Chromosome bands
q13.1
Genes
(Comprehensive set from GENCODE 19)
BRCA2-001 > protein coding
Exon 26/27 Forward
Exon 27 Forward
Intron 27 Forward
BRCA2-201 > protein coding
Exon 26/27 Reverse
Exon 27 Reverse
Intron 27 Reverse
Primer Legend
Detects both BRCA2-001/Short and BRCA2-201/Long
Detects only BRCA2-201/Long
c
Exon 26/27
Exon 27
Intron 27
ns
ns
ns
ΔΔCT
20
10
0
−10
Both Isoforms
BRCA2−001/Short Only
BRCA2−201/Long Only
BRCA2 Isoform Expressed
Tumor Type
Primary
Recurrence
Germline Mutation
BRCA1
BRCA2

Supplementary Figure 10. Validation of differential *BRCA2* isoform expression by quantitative reverse transcription PCR (RT-qPCR). A. RNA binding protein (ELAVL1/HuR and PABPC1) prediction for *BRCA2* transcripts based on RNA immunoprecipitation sequencing (RIP-seq) in GM12878 (Human B cells). ENST00000380152 refers to *BRCA2-001/Short*; ENST00000544455 refers to *BRCA2-201/Long*. B. Regions targeted by three sets of primers for detection of *BRCA2-001/Short* and *BRCA2-201/Long* by RT-qPCR. The regions were designed based on the RNA exome (capture-based) expression data. Color coding denotes whether primers were designed to detect both isoforms (black) or specifically *BRCA2-201/Long* (blue). C. Detection of *BRCA2* transcript regions by RT-qPCR, compared to the *BRCA2* isoform expression initially assessed by RNA-seq. Relative expression was normalized to *GAPDH* expression in one representative sample (n = 2 technical replicates from 20 biologically independent tumor samples in 14 patients; see **Supplementary Methods**)**.** Boxplot elements are as follows: median, center line; box limits, first and third quartiles (spanning the IQR, interquartile range); whiskers, 1.5x IQR in each direction; outliers plotted individually. Groupwise differences in expression were assessed separately for each primer set by Kruskal-Wallis test (α=0.05). NS = not significant.

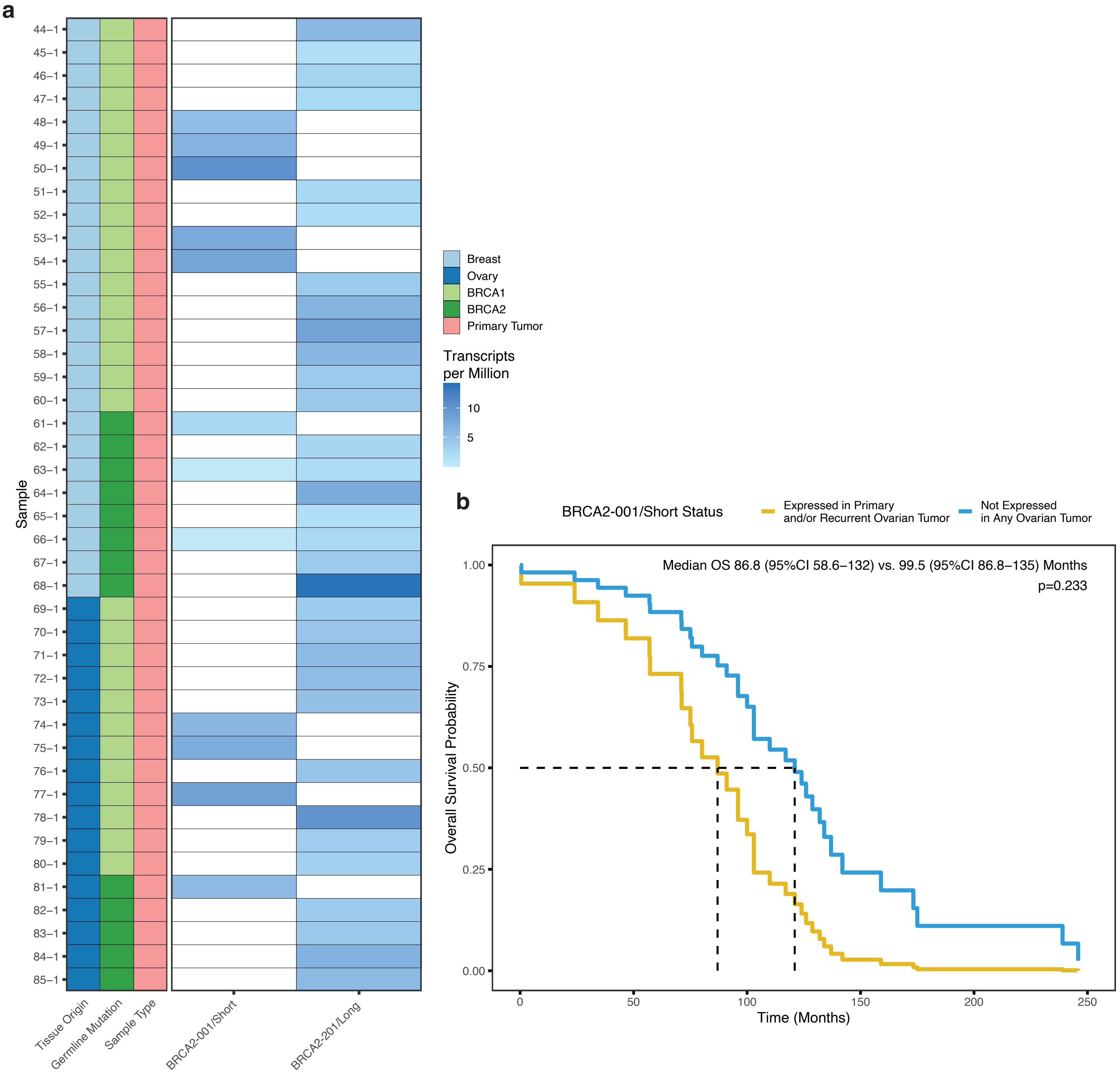
a
Sample
44–1
45–1
46–1
47–1
48–1
49–1
50–1
51–1
52–1
53–1
54–1
55–1
56–1
57–1
58–1
59–1
60–1
61–1
62–1
63–1
64–1
65–1
66–1
67–1
68–1
69–1
70–1
71–1
72–1
73–1
74–1
75–1
76–1
77–1
78–1
79–1
80–1
81–1
82–1
83–1
84–1
85–1
Tissue Origin
Germline Mutation
Sample Type
BRCA2-001/Short
BRCA2-201/Long
Breast
Ovary
BRCA1
BRCA2
Primary Tumor
Transcripts per Million
10
5
b
BRCA2-001/Short Status
Expressed in Primary and/or Recurrent Ovarian Tumor
Not Expressed in Any Ovarian Tumor
Median OS 86.8 (95%CI 58.6–132) vs. 99.5 (95%CI 86.8–135) Months
p=0.233
Overall Survival Probability
1.00
0.75
0.50
0.25
0.00
0
50
100
150
200
250
Time (Months)

Supplementary Figure 11. Differential isoform usage of *BRCA2* in a validation cohort of *BRCA1/2* mutation-associated primary tumors. A. *BRCA2* isoform usage by sample type in an independent cohort of 42 primary breast and ovarian tumors from *BRCA1/2* mutation carriers. B. Overall Survival (OS) curve for patients that expressed *BRCA2-001/Short* in any (primary or recurrent) ovarian tumor compared to those that did not. Survival proportions and p-value were calculated using a Cox proportional hazards model tested for significant associations with estrogen receptor status, age at diagnosis, *BRCA1* vs. *BRCA2* mutation, and tumor stage at diagnosis; and adjusted for patient recurrent status (α=0.05, see **Methods**).

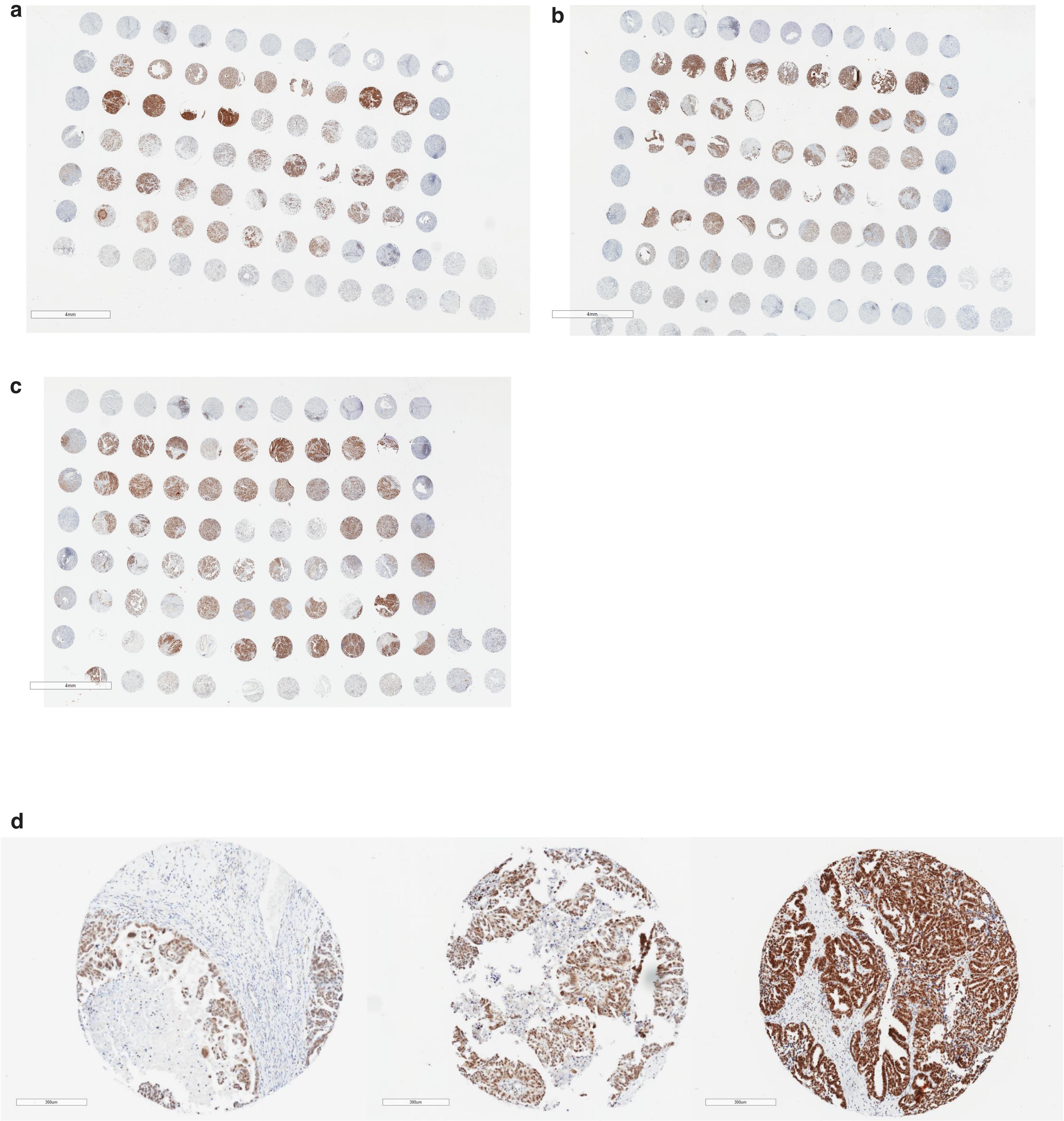
a
b
c
d
4mm
4mm
4mm
300um
300um
300um

Supplementary Figure 12. PARP1 expression in primary/recurrent tumors by immunohistochemistry (IHC). A. Image of PARP1 IHC in tissue microarray (TMA) of primary and recurrent breast tumors. B. Image of PARP1 IHC in TMA of primary and recurrent ovarian tumors (1/2). C. Image of PARP1 IHC in TMA of primary and recurrent ovarian tumors (2/2). For A-C, images were taken at 0.5x magnification. D. Representative images of individual TMA cores from ovarian tumors with low PARP1 expression (left, H-score = 125), medium PARP1 expression (middle, H-score = 200), and high PARP1 expression (right, H-score = 300). Images were taken at 7-7.2x. Images are representative of 23 total tumors for which PARP1 IHC was performed.

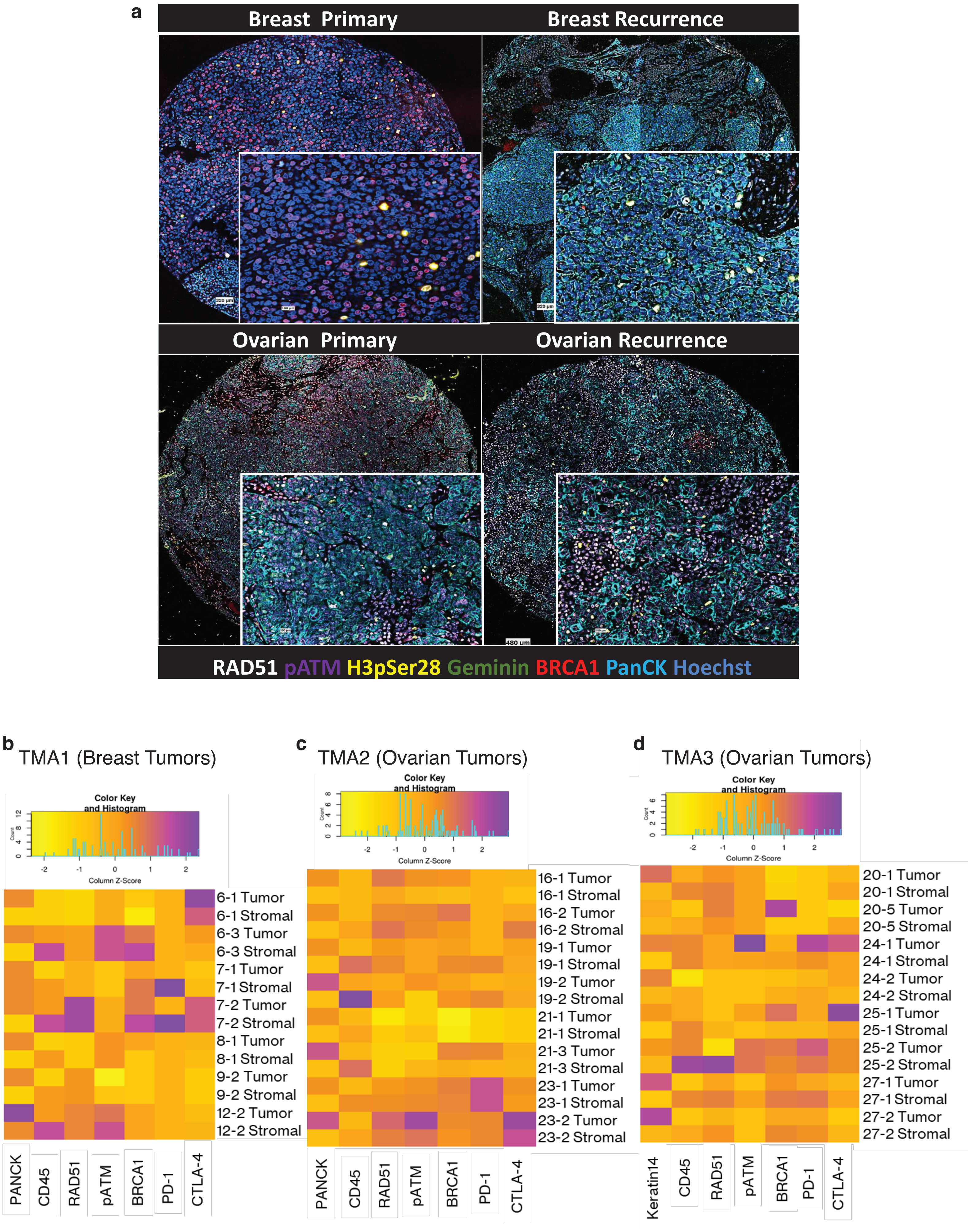
a
Breast Primary
Breast Recurrence
Ovarian Primary
Ovarian Recurrence
RAD51 pATM H3pSer28 Geminin BRCA1 PanCK Hoechst
b
TMA1 (Breast Tumors)
Color Key and Histogram
Column Z-Score
6-1 Tumor
6-1 Stromal
6-3 Tumor
6-3 Stromal
7-1 Tumor
7-1 Stromal
7-2 Tumor
7-2 Stromal
8-1 Tumor
8-1 Stromal
9-2 Tumor
9-2 Stromal
12-2 Tumor
12-2 Stromal
PANCK
CD45
RAD51
pATM
BRCA1
PD-1
CTLA-4
c
TMA2 (Ovarian Tumors)
Color Key and Histogram
Column Z-Score
16-1 Tumor
16-1 Stromal
16-2 Tumor
16-2 Stromal
19-1 Tumor
19-1 Stromal
19-2 Tumor
19-2 Stromal
21-1 Tumor
21-1 Stromal
21-3 Tumor
21-3 Stromal
23-1 Tumor
23-1 Stromal
23-2 Tumor
23-2 Stromal
PANCK
CD45
RAD51
pATM
BRCA1
PD-1
CTLA-4
d
TMA3 (Ovarian Tumors)
Color Key and Histogram
Column Z-Score
20-1 Tumor
20-1 Stromal
20-5 Tumor
20-5 Stromal
24-1 Tumor
24-1 Stromal
24-2 Tumor
24-2 Stromal
25-1 Tumor
25-1 Stromal
25-2 Tumor
25-2 Stromal
27-1 Tumor
27-1 Stromal
27-2 Tumor
27-2 Stromal
Keratin14
CD45
RAD51
pATM
BRCA1
PD-1
CTLA-4

Supplementary Figure 13. Comparisons of DNA damage markers and immune checkpoint proteins in paired primary/recurrent tumors by CODEX. A. Representative image of cell cycle and DNA damage markers in breast and ovarian primary and recurrent tumors. Images are representative of 23 tumors for which CODEX was performed. B. Expression of markers for cell type, DNA damage response, and immune checkpoints in primary and recurrent breast tumors from five patients. C. Expression of markers for cell type, DNA damage response, and immune checkpoints in primary and recurrent ovarian tumors from four patients. D. Expression of markers for cell type, DNA damage response, and immune checkpoints in primary and recurrent ovarian tumors from four additional patients. For B-D, tumor and stromal cells are displayed separately for each specimen; PANCK, Keratin14, and CD45 were used for gating of tumor and stroma (see **Supplementary Methods**). Tumors are numbered in chronological order following the convention [Patient] - [Tumor number] such that 16-1 is Patient 16's primary tumor, 16-2 the first recurrence, etc. For clinical metadata from individual tumors, see **Supplementary Data 1**. TMA = Tissue Microarray.

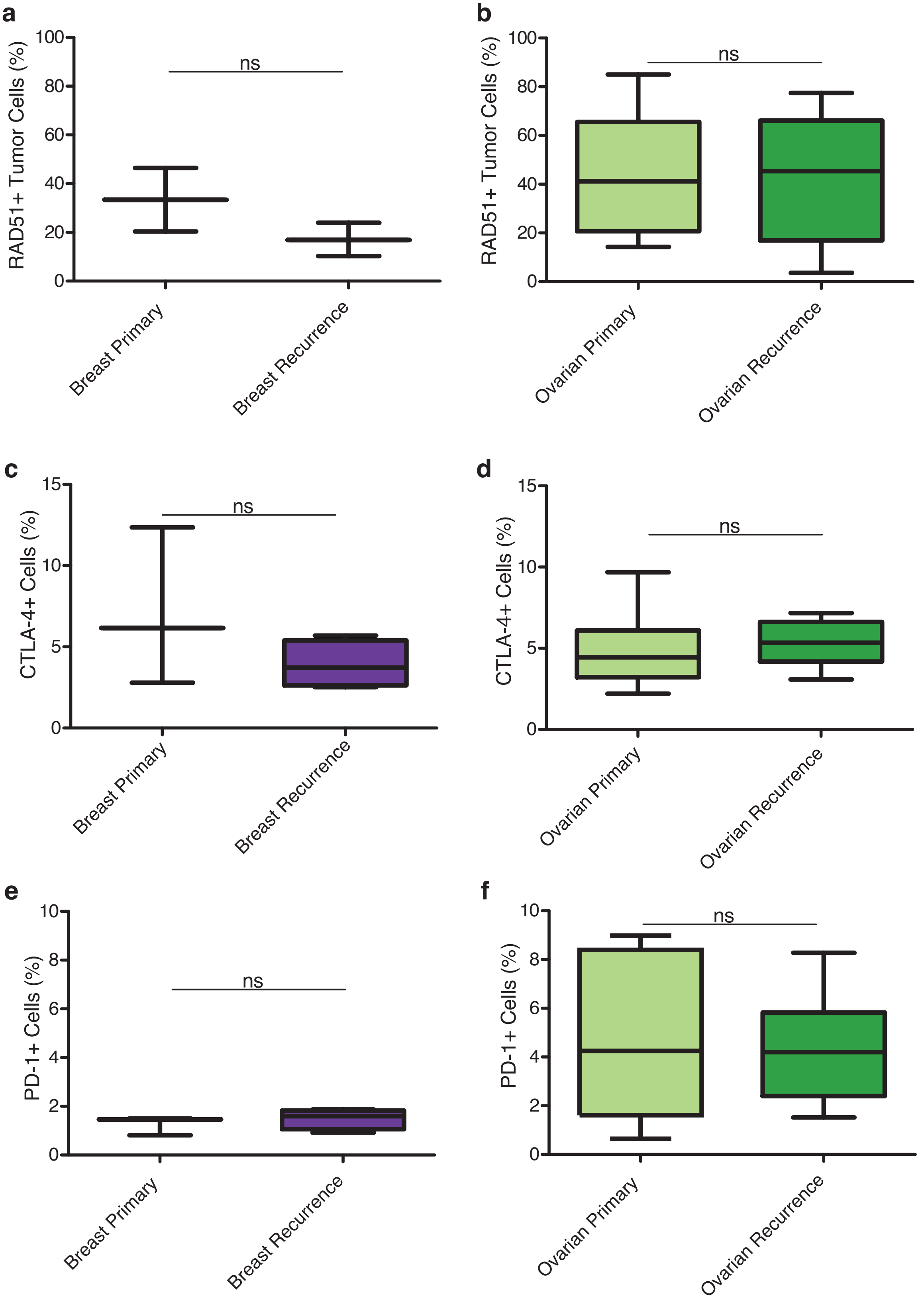

a
b
c
d
e
f
RAD51+ Tumor Cells (%)
CTLA-4+ Cells (%)
PD-1+ Cells (%)
ns
Breast Primary
Breast Recurrence
Ovarian Primary
Ovarian Recurrence

Supplementary Figure 14. Comparisons of RAD51 and immune checkpoint protein expression in primary and recurrent tumors by CODEX. A. Percent of RAD51+ tumor cells in primary vs. recurrent breast tumors. B. Percent of RAD51+ tumor cells in primary vs. recurrent ovarian tumors. C. Percent of CTLA-4+ cells in primary vs. recurrent breast tumors. D. Percent of CTLA-4+ cells in primary vs. recurrent ovarian tumors. E. Percent of PD-1+ cells in primary vs. recurrent breast tumors. F. Percent of PD-1+ cells in primary vs. recurrent ovarian tumors. For A-F, sample sizes were as follows: n=3 primary breast tumors, n=4 recurrent breast tumors, n=8 primary ovarian tumors, n=8 recurrent ovarian tumors. Boxplot elements are as follows: median, center line; box limits, first and third quartiles (spanning the IQR, interquartile range); whiskers, 1.5x IQR in each direction; outliers plotted individually. Groupwise differences were determined by two-sided Wilcoxon rank sum test (α=0.05). NS = not significant.

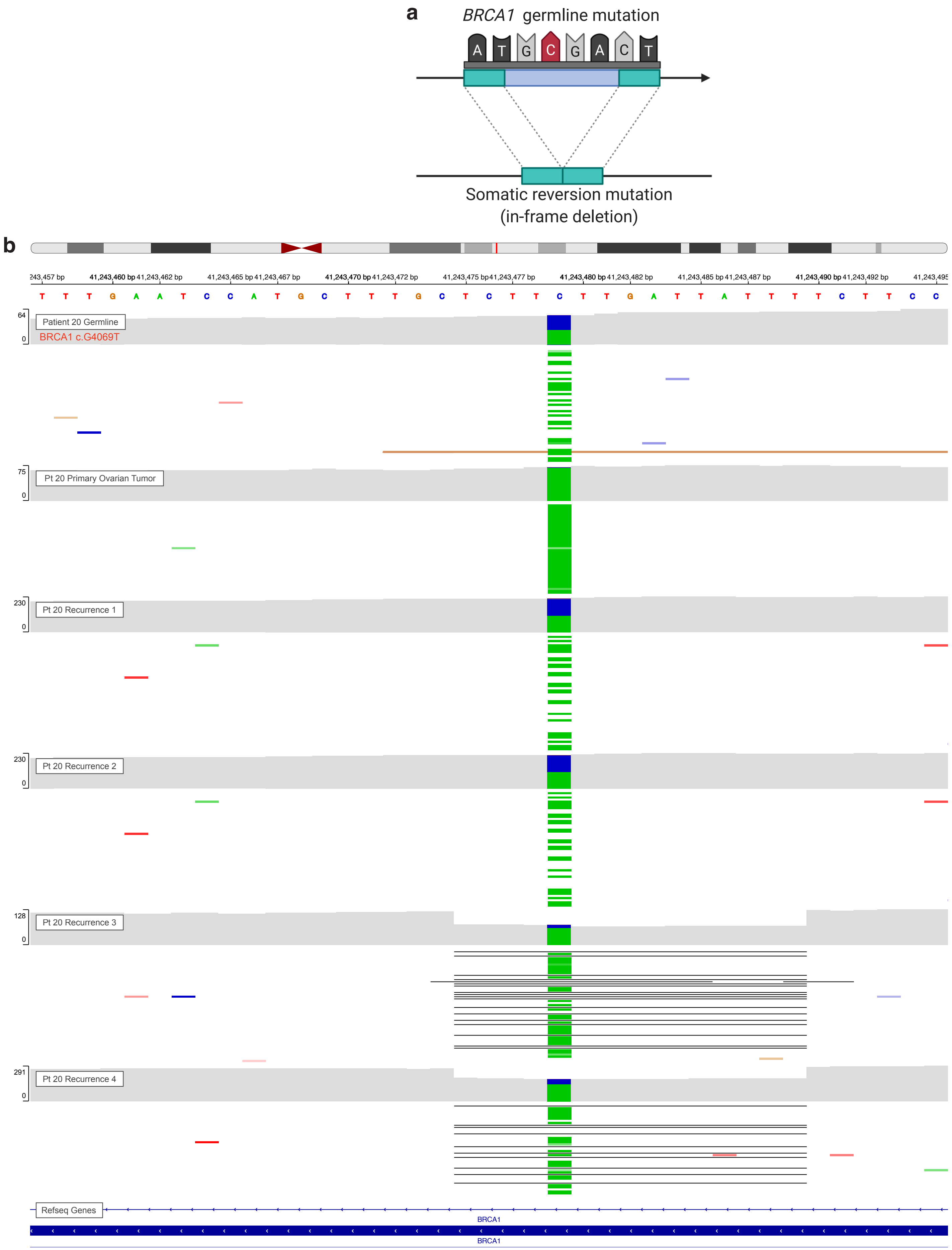
a
BRCA1 germline mutation
A T G C G A C T
Somatic reversion mutation
(in-frame deletion)
b
Patient 20 Germline
BRCA1 c.G4069T
Pt 20 Primary Ovarian Tumor
Pt 20 Recurrence 1
Pt 20 Recurrence 2
Pt 20 Recurrence 3
Pt 20 Recurrence 4
Refseq Genes
BRCA1
BRCA1

Supplementary Figure 15. Somatic reversion of *BRCA1* after PARPi in Patient 20. A. Graphical representation of in-frame somatic deletion of germline nonsense mutation in *BRCA1* (mutation pictured is not Patient 20's actual germline mutation). B. Integrative Genomics Viewer (IGV) tracks from whole exome sequencing of germline and tumor DNA from patient 20. For each sample, forward strand alignment and coverage tracks are displayed around the site of the patient's germline *BRCA1* c.G4069T (p.E1357X) mutation. G>T mutation appears as C>A because *BRCA1* lies on reverse strand. Pt = patient.
