# Supplementary material for: Analysis of matched primary and recurrent BRCA1/2 mutation-associated tumors identifies recurrence-specific drivers
Source: Nat Commun. 2022 Nov 7;13:6728. doi: 10.1038/s41467-022-34523-y (PMC9640723; doi:10.1038/s41467-022-34523-y)
Supplement: Supplementary file 2 — Description of Additional Supplementary Information [file 41467_2022_34523_MOESM2_ESM.pdf]

Description of Additional Supplementary Files

Supplementary Data1: Clinical metadata and sequencing performed by tumor (Basser cohorts)

Supplementary Data 2: Pathogenic mutations by sequencing type and by tumor group (primary-private, recurrence-private, and shared)

Supplementary Data 3: Pathogenic TP53 mutations by sequencing type and tumor group

Supplementary Data 4: Tumor mutational burden, HRD, and aneuploidy scores by tumor

Supplementary Data 5: Gene set enrichment analysis from whole exome sequencing (mutations and copy number variation)

Supplementary Data 6: MutSigCV results from primary and recurrent tumor cohorts

Supplementary Data 7: Targeted sequencing metrics, summary and for BRCA1 and BRCA2 genes

Supplementary Data 8: Somatic Loss of Function Mutations in BRCA1/2 by sequencing type and tumor group

Supplementary Data 9: Results from all GISTIC analyses

Supplementary Data 10: Subtractive analysis of GISTIC segments in primary vs. recurrent tumors

Supplementary Data 11: PARP1 copy number in primary/recurrent and TCGA cohorts

Supplementary Data 12: TCGA tumors by group

Supplementary Data 13: Gene set enrichment analysis from RNA sequencing

Supplementary Data 14: Gene fusions (FusionInspector output) involving MALAT1 and immunoglobulin genes

Supplementary Data 15: BRCA2 isoform expression by tumor and clinical metadata used for survival analyses; Cox Proportional Hazards models

Supplementary Data 16: RT-qPCR results from validation of differential BRCA2 isoform expression; primer sequences
